# Composite Bifunctional Electrocatalyst for the Oxygen Reduction and Evolution Reactions

**DOI:** 10.1021/acsmaterialsau.5c00034

**Published:** 2025-07-03

**Authors:** Casey E. Beall, Emiliana Fabbri, Juliana Bruneli Falqueto, Sebastian Siegrist, Jinzhen Huang, Natasha Hales, Dominika Baster, Mario El Kazzi, Sayaka Takahashi, Yuto Shirase, Makoto Uchida, Thomas J. Schmidt

**Affiliations:** † 28498 PSI Center for Energy and Environmental Science, 5232 Villigen PSI, Switzerland; ‡ Institute for Molecular Physical Science, ETH Zürich, 8093 Zürich, Switzerland; § Hydrogen and Fuel Cell Nanomaterials Center, 38146 University of Yamanashi, 400-0021 Kofu, Japan

**Keywords:** oxygen reduction reaction, oxygen evolution reaction, unified regenerative fuel cells, composite bifunctional electrocatalyst, AEMWE, AEMFC

## Abstract

Bifunctional oxygen electrocatalysts are subjected to stringent performance and stability criteria. The catalyst must achieve high oxygen evolution reaction (OER) activity while in electrolyzer operation, as well as high oxygen reduction reaction (ORR) activity while in fuel cell operation. Additionally, the catalyst must be stable over a wide potential range and withstand alternating reducing and oxidizing potentials. In this work, a composite Ni_0.95_Fe_0.05_O_1±δ_/NiCo_2_O_4_ is rigorously tested as a bifunctional catalyst for anion exchange membrane (AEM) fuel cell and electrolyzer operation. An alternating potential stability test is performed, which unveils the areas where the bifunctional catalyst needs improvement. The OER activity of the catalyst is not hindered by the harsh conditions. However, the ORR activity deteriorates. Both the fundamental rotating disk electrode (RDE) methodology and AEM single-cell testing are used to evaluate the electrode activity and stability. The difference in results between the two techniques emphasizes the importance of evaluating the catalyst under applied conditions. The results of this study provide guidance for the development of new high-performing bifunctional catalysts.

## Graphical abstract

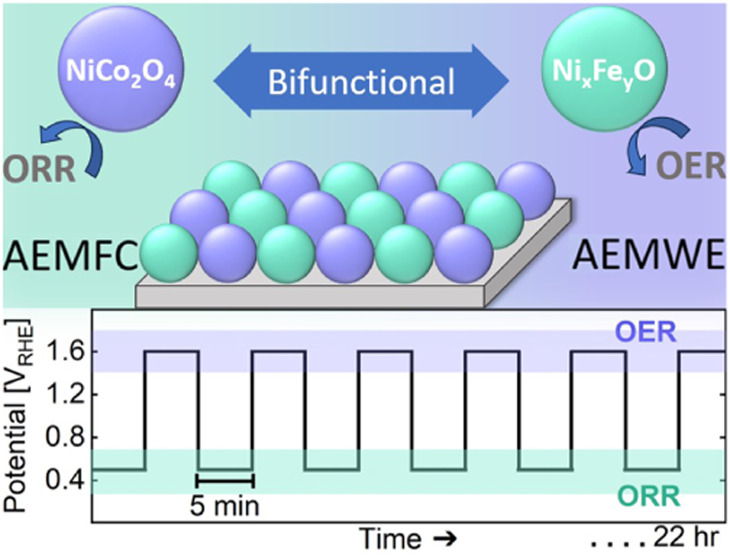

## Introduction

The increasing global consumption of energy has necessitated the growth of environmentally friendly energy conversion and storage devices.^1,2^ Hydrogen is a promising energy carrier due to its high gravimetric energy density and the potential for zero emissions when used in a fuel cell or electrolyzer.^3,4^ Unified regenerative fuel cells (URFC) operate as either a hydrogen fuel cell or electrolyzer, depending on if there is an energy shortage or excess. The advantage of URFCs is that only one device is required instead of a separate fuel cell and electrolyzer, resulting in lower costs and a system with reduced weight and volume.^5,6^ Therefore, URFCs are particularly valuable for confined spaces and remote areas, for example, aerospace and transportation applications.^5,7^ The bifunctional oxygen electrode will undergo the oxygen reduction reaction (ORR) when the URFC operates as a fuel cell and will undergo the oxygen evolution reaction (OER) when the URFC operates as an electrolyzer. ORR and OER both suffer from high overpotentials and sluggish kinetics, resulting in efficiency losses.^8,9^ Additionally, the stability of the bifunctional catalyst is also a challenge due to the wide potential range and alternating potential conditions that occur in a URFC.^10–14^ Currently, one of the main impediments to URFC advancement is the activity and stability of the bifunctional oxygen electrocatalyst.^13,15,16^

Our previous work investigated various methods for designing bifunctional catalysts.^17^ We found the most successful method was combining an ORR catalyst with an OER catalyst into a composite consisting of a homogeneous mixture of the two catalysts. Other studies have also investigated various composite design strategies such as a layered structure, a homogeneous mixture, or a core–shell design.^10–12,18–21^ In this study, an ORR catalyst is mixed with an OER catalyst in a 50/50 wt % ratio to form a homogeneous catalyst layer. In order to select one ORR and one OER catalyst for the composite, a wide variety of non-noble metal catalysts are first screened for activity and stability. Then, the most active ORR catalyst, NiCo_2_O_4_ (NiCo), is combined with the most active OER catalyst, Ni_0.95_Fe_0.05_O_1±δ_ (NiFe), into the composite Ni_0.95_Fe_0.05_O_1±δ_/NiCo_2_O_4_ (NiFe/NiCo).

Spinels (AB_2_O_4_) are promising ORR and OER catalysts due to their mixed valence states, the ability to tune their properties through A and B-site substitution, and their non-noble metal composition.^22–25^ Previous studies have investigated NiCo primarily as an ORR catalyst, often as a composite with a carbon such as N-doped graphene.^26–30^ Additionally, NiCo has been occasionally studied as an OER catalyst, often with doping or modification, and as a bifunctional catalyst.^31–35^ Ni–Fe oxides are well-known to be highly active OER catalysts.^36–38^ Doping Ni oxide with ∼5–50 atom % Fe increases the OER activity of the catalyst significantly.^36,37,39^ The structural versatility of Ni–Fe-based perovskites and spinels allows for the fine-tuning of properties that can influence the binding energies of oxygen intermediates, a key factor for optimizing OER and ORR activity. An example of a property that can be tuned in Ni–Fe-based materials is the mixed valence states, which can significantly change the bond strength between surface sites and intermediate species.^22,39^ Additionally, their chemical stability in alkaline environments, combined with the earth-abundant nature of Ni and Fe, makes these materials promising candidates for scalable and sustainable catalyst development.^22,36,38^ A few studies have also investigated their bifunctional activities.^22,40^

The thin-film rotating disk electrode (RDE) methodology is a useful technique for determining the intrinsic electrocatalytic activity of catalysts without the uncertain interference of water management effects, mass transport resistances, and catalyst utilization.^41,42^ Additionally, the protocol for RDE measurements can usually be performed in a shorter time frame than in membrane electrode assembly (MEA) single-cell tests. Therefore, RDE is often used to assess the catalytic activity of possible catalysts. In this study, both RDE and single-cell electrolyzer and fuel cell measurements are completed, in order to assess both the intrinsic activity and the activity under real conditions. Additionally, the results from the two methods are compared.

To investigate composite NiFe/NiCo as a bifunctional oxygen electrocatalyst, the initial ORR and OER activity of the catalyst is measured using RDE. Then, the catalyst is subjected to an alternating potential stability protocol to simulate an URFC switching between fuel cell and electrolyzer operation. The individual catalysts NiCo and NiFe are measured separately in RDE as references, as well as the composite with carbon black (NiFe/NiCo/CB). Conductive carbon supports are widely used in electrocatalysis due to their high surface area, good electrical conductivity, and ability to disperse catalytic nanoparticles. These properties enhance electron transport within the electrode material, thereby improving the catalytic activity for both OER and ORR. It is well-known that carbon corrosion occurs in an electrolyzer, leading to performance losses or even cell failure.^43–45^ Therefore, carbon should be avoided in the oxygen bifunctional catalyst layer. However, it is found in this study that carbon plays an essential role in ORR. Therefore, carbon is added to the catalyst layer of the composite NiFe/NiCo as a comparison and to further clarify the influence of carbon on bifunctional activity. The results highlight the properties required for high-performing bifunctional catalysts.

Single-cell anion exchange membrane fuel cell (AEMFC) and water electrolyzer (AEMWE) tests are performed to assess the catalysts under more applied conditions. This investigation clearly shows the challenges of bifunctional catalyst design. The composite excels at water electrolysis, indicating that a mixed electrode with two catalysts is a solid strategy. However, the fuel cell performance of the ORR catalyst suffers when mixed with an OER catalyst. Additionally, the composite is unable to reach high current densities in AEMFC without the addition of carbon. A discussion is made on the contrasting results achieved in RDE *vs* single-cell testing. Finally, recommendations are made for future bifunctional catalyst design and testing procedures.

## Results and Discussion

Based on our previous findings,^17^ the most effective strategy for developing bifunctional catalysts involved combining separate ORR and OER catalysts into a homogeneous composite. To identify suitable candidates, a wide variety of catalyst materials were screened with RDE in order to select the most active and stable ORR and OER catalysts to combine into a bifunctional composite electrode with a 50/50 wt % ratio for this study. The screening protocol involved measuring the initial ORR and OER activity of various catalysts, including perovskites, spinels, layered double hydroxides (LDH), and metal oxides. Results for less promising samples, along with further details of the screening protocol, are provided in Figure S1. Additionally, for the most promising samples, activity measurements were performed both before and after stability testing. After the screening protocol, the most active catalysts identified were NiFe for OER and NiCo for ORR. These two catalysts were subsequently selected to create a bifunctional composite electrode, NiFe/NiCo. The NiFe/NiCo composite consisted of 50 wt % NiFe and 50 wt % NiCo. To produce the composite electrodes, the two catalysts were mixed homogeneously in an ink and either drop-casted or spray-coated onto a substrate. Hence, the single material electrode and the composite electrode undergo the same preparation process, avoiding material alteration during the mixing process.

NiCo was synthesized using a hydrothermal method followed by calcination. The resulting material crystallized into a spinel phase (Figure S2a) with particles of platelet or wire-like morphology forming a micrometer-sized agglomerate (Figure 1a). NiFe was synthesized using a flame-spray method described previously.^46,47^ The oxide has a cubic crystal structure (Figure S2b) and a feather-like morphology with smaller nanoparticles than NiCo (Figures 1b). Additional details are provided in the Supporting Information (SI), Figures S3–S6, which include SEM and EDX mapping analyses of the catalyst powders (NiCo and NiFe) and catalyst composites (NiFe/NiCo and NiFe/NiCo/CB). EDX mapping analysis of the NiCo powder showed a consistent distribution of Ni and Co across the sample (Figure S3). The atomic composition was found to be approximately 18% for Ni and 31% for Co, resulting in a Ni:Co atomic ratio of approximately 0.58:1. While slightly deviating, this ratio is reasonably consistent with the expected 0.50:1 ratio for the nominal NiCo_2_O_4_ composition. For the NiFe sample (Figure S4), the EDX mapping revealed a homogeneous distribution of Ni and Fe across the surface. The atomic composition was found to be approximately 46% Ni and 4% Fe. This ratio corresponds well with the nominal stoichiometry of Ni_0.95_Fe_0.05_O_1±δ_, where Fe represents 5 at. % of the total metal content (defined as Ni + Fe). The slight deviation (Fe ≈ 8% detected by EDX) is within the expected measurement uncertainty for EDX (±2% error) and supports the successful incorporation of Fe into the Ni-based oxide. For the composite samples (Figures S5 and S6), the elemental composition confirmed the presence of Ni, Co, and Fe, with nickel being the most abundant and Fe the least abundant metal species detected. Additionally, it is important to point out that the aluminum signal comes from the aluminum substrate of the SEM stubs. Using Brunauer–Emmett–Teller (BET) analysis, the surface area of the two catalysts was estimated. NiFe has a slightly higher surface area (39 m^2^/g) than NiCo (23 m^2^/g). Additionally, the electrical conductivities of the two nanopowders were estimated using ex-situ 4-wire impedance spectroscopy measurements (Table S1). NiCo exhibited an almost metallic conductivity. In comparison, NiFe was 6 orders of magnitude more resistive than NiCo. The structural and compositional data confirm that the synthesized oxides exhibit the desired crystal structures, nanoscale particle sizes with high surface areas, and uniform elemental distribution. While NiFe presents a higher surface area, which can favor oxygen reactions by increasing the probability of having accessible active sites, the higher electrical conductivity of NiCo may favor better ORR performance. These complementary characteristics support their application in bifunctional electrocatalysis.

**Figure 1:**
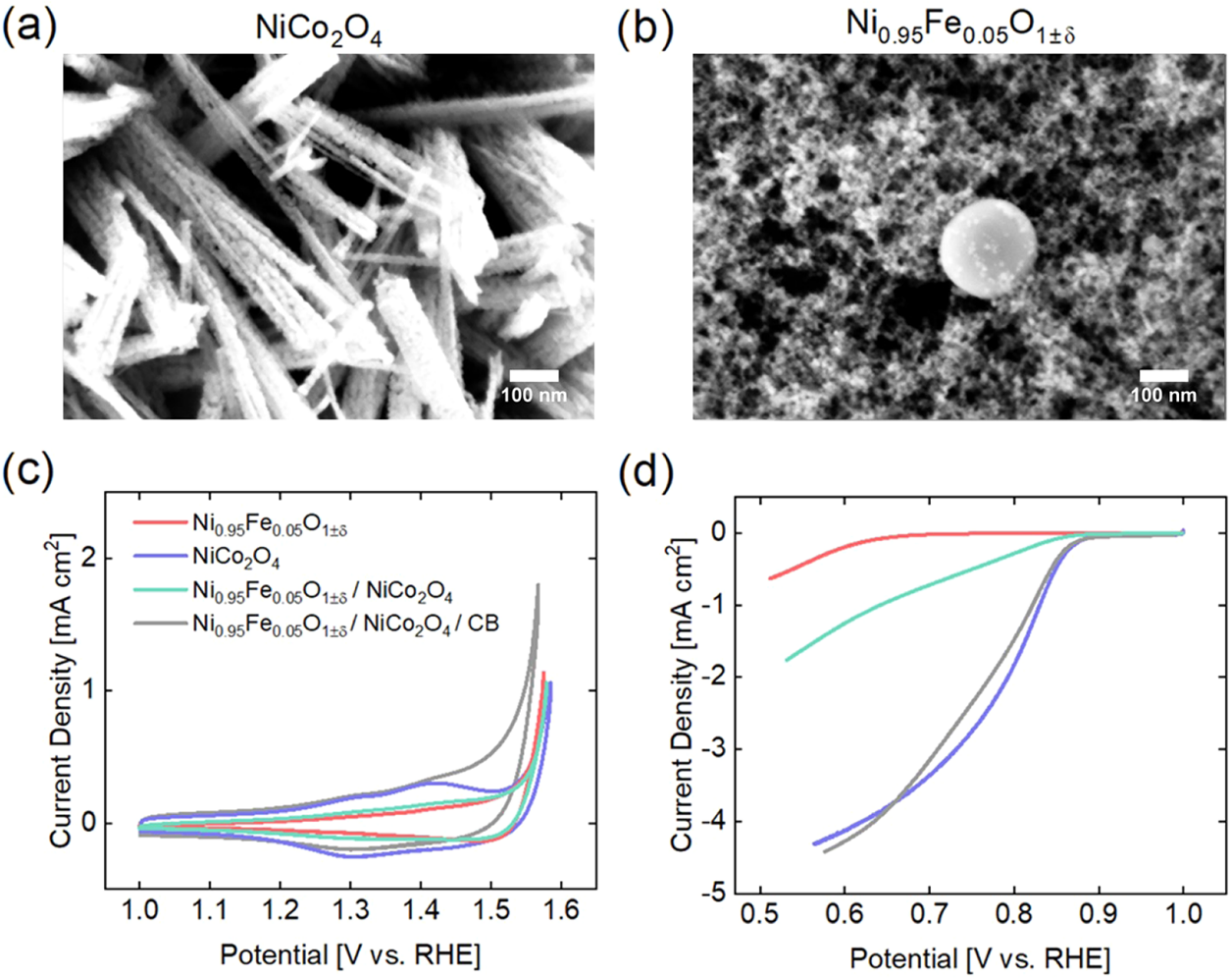
As-prepared nanopowders (a) NiCo and (b) NiFe were imaged with SEM to demonstrate their nanostructure. The (c) OER and (d) ORR activities of the single phase and composite materials were measured with RDE in oxygen-saturated 0.1 M KOH with a rotation rate of 1600 rpm and scan rate of 5 mV s^–1^. Three CVs were performed and the third sweep is shown here (cathodic for ORR).

NiFe and NiCo samples and their composites were further analyzed using X-ray photoelectron spectroscopy (XPS). The XPS survey, along with the Ni 2p, Co 2p, O 1s, and C 1s spectra, are shown in Figure S7. The analysis of the Ni spectra was conducted following the methodology outlined in Biesinger et al.^48^ A comparison of the spectral fitting parameters for the XPS spectra of Ni 2p3/2 core level peaks of NiFe, NiCo samples, and their composites with the fitting parameters of NiFe_2_O_4_ in the literature (see Tables S2 and S3), suggests that Ni at the surface is predominantly in the 2+ oxidation state in all samples and composites. However, the presence of Ni^3+^ cannot be completely excluded based on acquired XPS spectra at the Ni 2p core level. The Co 2p core level spectra indicate a mixed 2+/3+ oxidation state of Co at the surface of NiCo and the composite NiFe/NiCo, similarly to Co_3_O_4_ reported in the literature.^48^ The Fe signal could not be analyzed due to its low concentration in the NiFe sample and the overlap of the Ni Auger peak in the Fe 2p spectral region.

### Bifunctional Catalytic Activity in RDE

The catalytic and redox activities of the single materials and composites were evaluated using cyclic voltammetry (CV) in RDE, and the results are shown in Figure 1c,d. In Figure 1c, between 1.25 and 1.45 V, several redox peaks are apparent in the CVs of NiCo and NiFe/NiCo/CB. These most likely include contributions from the Co^2+^/Co^3+^ and/or Ni^2+^/Ni^3+^ transitions.^49^ No Ni^2+^/Ni^3+^ redox peaks are observable for NiFe, consistent with previous reports.^39^ Interestingly, in NiFe/NiCo, without the addition of carbon, no redox couples are visible in the related CV shown in Figure 1c, possibly due to the lower electronic conductivity of the composite material. The OER activities (Figure 1c) of the materials without carbon are remarkably similar, while the composite with carbon has higher OER activity than the other samples. Contrarily, the ORR activities (Figure 1d) of the four samples are distinctly different. NiFe has little to no activity for oxygen reduction. NiCo greatly outperforms NiFe and has a significantly earlier onset. The composite NiFe/NiCo without carbon presents slightly less ORR activity than the average of its counterparts (NiFe and NiCo). This decrease in activity is most likely due to the lack of electrical conductivity of NiFe, which isolates the ORR active sites of NiCo. Then, when carbon is added (NiFe/NiCo/CB), the ORR activity equals that of NiCo. Carbon has been shown previously to increase the ORR activity of transition metal oxides through either increased electrical conductivity pathways and/or a synergistic reaction mechanism.^50,51^ While it is well-known that carbon corrosion occurs in an electrolyzer, leading to performance losses or even cell failure,^43–45^ it is also widely reported that Ni-based OER catalysts improve their activity when they initially operate in the OER potential region due to surface reconstruction.^36,39^ Therefore, the decrease in activity due to carbon corrosion in the OER potential region can be masked by the increase in the catalyst OER activity due to surface reconstruction.

### Stability Protocol in RDE

In Figure 1, the catalysts’ ORR activities were measured separately from their OER activities, each time with a newly drop-casted catalyst thin film. Therefore, the catalyst was exposed to either reducing or oxidizing potentials. However, under operation in a URFC, the catalyst will undergo both ORR and OER within a wide potential range. To assess how the catalyst would respond to switching between electrolyzer and fuel cell operation, an alternating chronoamperometry stability protocol was applied and the results are given in Figure 2. The potential was alternated between 1.6 V (OER) and 0.5 V (ORR) for 22 h, holding for 5 min at each potential. The step duration of 5 min was chosen to ensure the current reached a plateau. The last 20% of each potential step was averaged and plotted as one point in Figure 2b–e.

**Figure 2:**
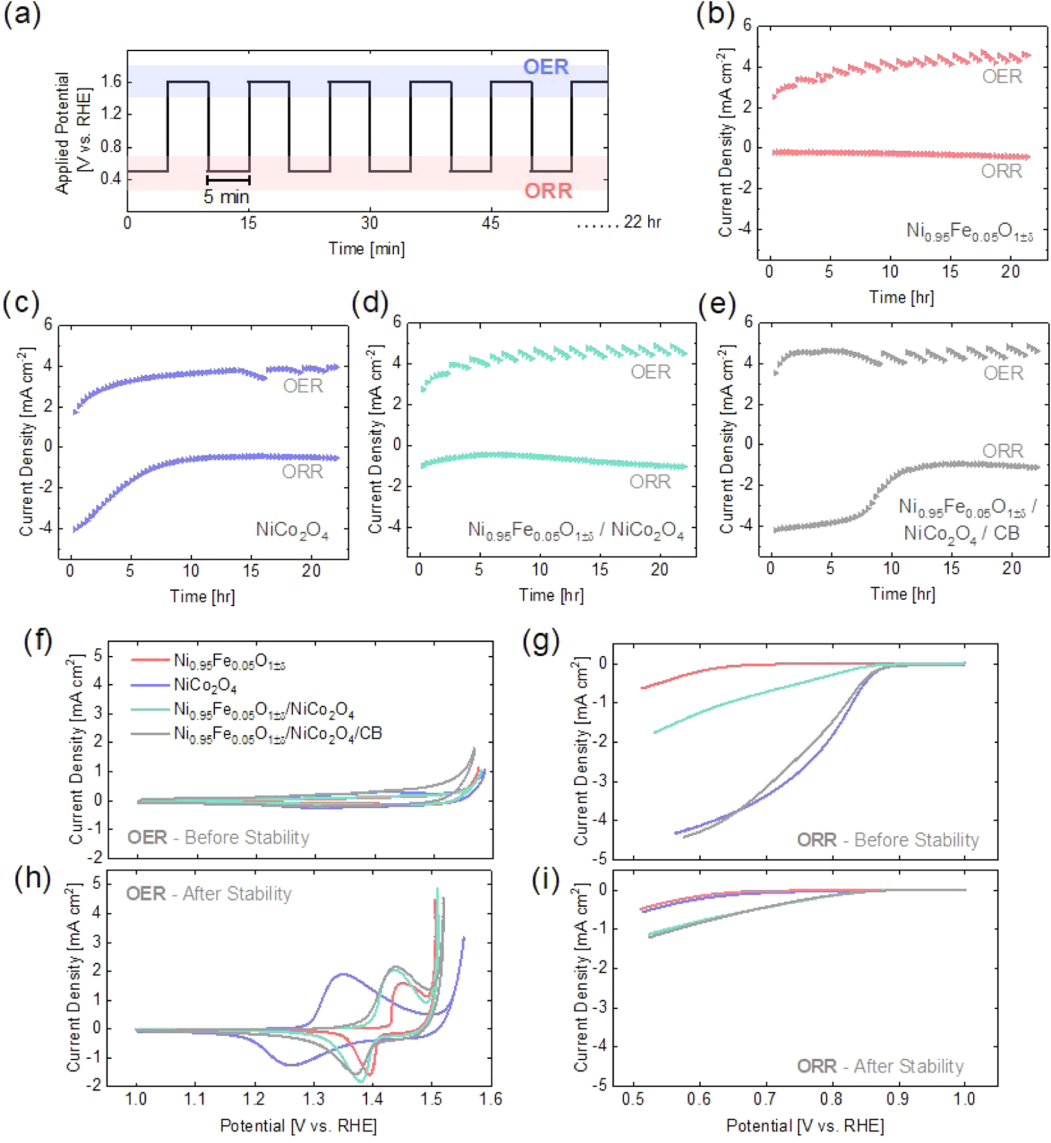
Stability protocol performed using RDE in 0.1 M KOH with a rotation rate of 1600 rpm. (a) Chronoamperometry is performed where the potential is alternated between 1.6 and 0.5 V, holding 5 min at each potential step for 22 h. The last 20% of each step is averaged and is plotted as one point for (b) NiFe, (c) NiCo, (d) NiFe/NiCo, and (e) NiFe/NiCo/CB. The (f) OER and (g) ORR activities of the samples were measured using cyclic voltammetry before starting the stability protocol. Then, the (h) OER and (i) ORR activities were measured again after the stability protocol. Three CV cycles were performed and the third cycle is shown in (f–i), the cathodic scan for ORR.

The positive current in Figure 2b–e corresponds to the chronoamperometry measurements at the applied potential of 1.6 V, where OER occurs. The oscillating pattern observable in the OER current of every material is most likely due to the formation and subsequent release of bubbles during OER. The current produced during OER for NiFe/NiCo/CB is relatively constant over time, with a slight increase at the beginning of the protocol. The other materials without carbon increase in OER activity throughout the duration of the protocol, until around approximately 15 h when a plateau is reached and the current stabilizes. This increase in OER activity observed for all materials is most likely a self-assembled amorphous oxyhydroxide layer formed through cation dissolution and redeposition under oxidative potentials.^52–54^ Ni, Co, and Fe containing metal oxides have been reported to form a Ni, Co, and/or Fe containing oxyhydroxide layer during OER.^53,55–57^ The dynamic formation of this surface layer usually leads to higher OER activity of the electrocatalyst^58,59^ and would explain the increase in OER activity observed over time. However, the surface reconstruction appears to reach a state of equilibrium around 10–15 h, as evidenced by the stabilization in the measured OER current.

The alteration of the oxide surface is further illustrated by observing the change in redox activity before and after alternating potential testing (Figure 2f,h). Cyclic voltammetry measurements from 0–1.6 V were performed with RDE before and after the stability protocol and are shown in Figure 2f,h, and in more detail in Figure S8. Before the stability test, NiFe and NiFe/NiCo exhibited no clear redox peaks. After the stability test, NiFe, NiFe/NiCo, and NiFe/NiCo/CB exhibit a set of redox peaks with a half-wave potential (*E*_1/2_) of approximately 1.4 V, with NiFe having a slightly higher *E*_1/2_. At oxidative potentials, prior to the onset of OER, Ni-based catalysts will form Ni­(OH)_2_ and at higher potentials NiOOH is formed by the reaction in eq 1.^60–62^

1 $${\mathrm{Ni}\left(\mathrm{OH}\right)}_{2}+OH^{-}\to \mathrm{NiOOH}+H_{2}O+e^{-}$$

This transformation of α-Ni­(OH)_2_ or β-Ni­(OH)_2_ to γ-NiOOH (Ni^3.3+^–Ni^3.67+^) or β-NiOOH (Ni^2.7+^–Ni^3.0+^), respectively, results in a change in Ni oxidation state.^57,63,64^ Therefore, the redox peaks of the NiFe-containing samples in Figure 2h are most likely attributable to the Ni­(OH)_2_/NiOOH transformations, which have been shown to occur at these potentials.^36,37,60^ It is quite likely that the catalyst layer may contain a mixture of species, as previous work has shown that the transformation between Ni­(OH)_2_ and NiOOH species is not fully reversible and mixed phases have been identified.^60,64^

Initially, NiCo has two anodic redox peaks at 1.3 and 1.4 V and one cathodic peak at 1.3 V (Figure S9). After the stability protocol, NiCo presents only one anodic peak at 1.35 V and one cathodic peak at 1.26 V (Figure 2h). The redox peaks can most likely be assigned to overlapping Ni and Co redox transitions. Overall, the redox activity of NiCo significantly increased after stability testing. The simultaneous decrease in ORR activity and increase in OER activity, as well as the increased redox activity of NiCo, would suggest a surface reconstruction similar to a self-assembled oxyhydroxide. An oxyhydroxide surface layer occurs for many Ni- and Co-based oxides during OER.^53,55–57^

During the stability protocol (Figure 2a), the potential was alternated to 0.5 V, where ORR is actively occurring, and the resulting negative current is plotted in Figure 2b–e for each material. NiFe is the least active of the studied materials and experiences little to no change in ORR activity over the protocol. NiCo experiences a continuous decrease in ORR current within the first 10 h before reaching a steady current density. Interestingly, a semiplateau in OER current is reached around this time as well. The composite NiFe/NiCo slightly decreases in ORR activity for the first 5 h before reversely increasing in ORR activity for the remainder of the protocol. Similar to NiCo, NiFe/NiCo/CB reaches a steady state around 10 h. However, for NiFe/NiCo/CB the decrease is logistic rather than linear; until hour 10, the ORR activity is mostly steady. Also, around hour 10, the growth and release of bubbles becomes apparent in the OER current and may have some impact on the ORR activity.

The change in ORR activity due to the stability protocol is further elaborated in Figure 2g,i. Linear sweep voltammetry measurements with RDE were performed from 1.0–0.5 V before and after the chronoamperometry stability protocol. After alternating potential testing, NiFe/NiCo and NiFe/NiCo/CB have the highest ORR activity. NiCo and NiFe/NiCo/CB undergo significant degradation in ORR performance. Our previous studies have shown that the formation of a (Co/Fe)­O­(OH) layer on bifunctional catalyst Ba_0.5_Sr_0.5_Co_0.8_Fe_0.2_O_3±δ_ (BSCF) and other perovskites during OER hinders the ORR activity of the perovskite.^17,65,66^ Although the nature of the surface transformation of the NiCo surface during stability testing is not as clear, the evolution of the redox activity during the alternating stability protocol can presumably be related to the surface transformation or possibly transition metal leaching, with consequent ORR activity decrease. Carbon corrosion is known to occur at OER potentials^43,67^ and, therefore, most likely also plays a role in the decrease in ORR activity for NiFe/NiCo/CB.

### AEMFC and AEMWE Single-Cell Tests

The feasibility of the bifunctional catalysts in a URFC was further assessed with AEMFC and AEMWE single-cell tests. The fuel cell and electrolyzer tests were performed separately. However, the oxygen electrode catalyst layers were the same for both devices (*i*.*e*., catalyst loading and ionomer concentration), to emulate a URFC. The anion exchange membrane was an ammonium-containing copolymer (QPAF-4), previously described by Ono *et al*.,^68^ synthesized in-house with high hydroxide ion conductivity and excellent mechanical properties and chemical stability. The most promising bifunctional catalyst from the RDE assessment was NiFe/NiCo. Therefore, this sample was chosen as the primary focus of the single-test measurements. NiFe/NiCo/CB was also tested as a comparison to understand the effect of carbon.

The catalysts’ initial performance in an electrolyzer was assessed through polarization curves (Figure 3a). NiFe was measured as a reference to compare the activity of a single OER catalyst with a mixed composite in AEMWE. All three catalysts, NiFe, NiFe/NiCo, and NiFe/NiCo/CB, exhibit excellent electrolysis performance and outperform the reference commercial IrO_2_ catalyst at high current densities (more details in the SI, Figure S10). NiFe is the most active catalyst and NiFe/NiCo/CB is the least active, although the difference in activity between NiFe/NiCo and NiFe/NiCo/CB is slight. When comparing the single OER catalyst (NiFe) to the composites, the substitution of 50 wt % NiCo only slightly increases the cell voltage. Therefore, a mixed composite of OER and ORR catalysts is a successful strategy for electrolysis.

**Figure 3:**
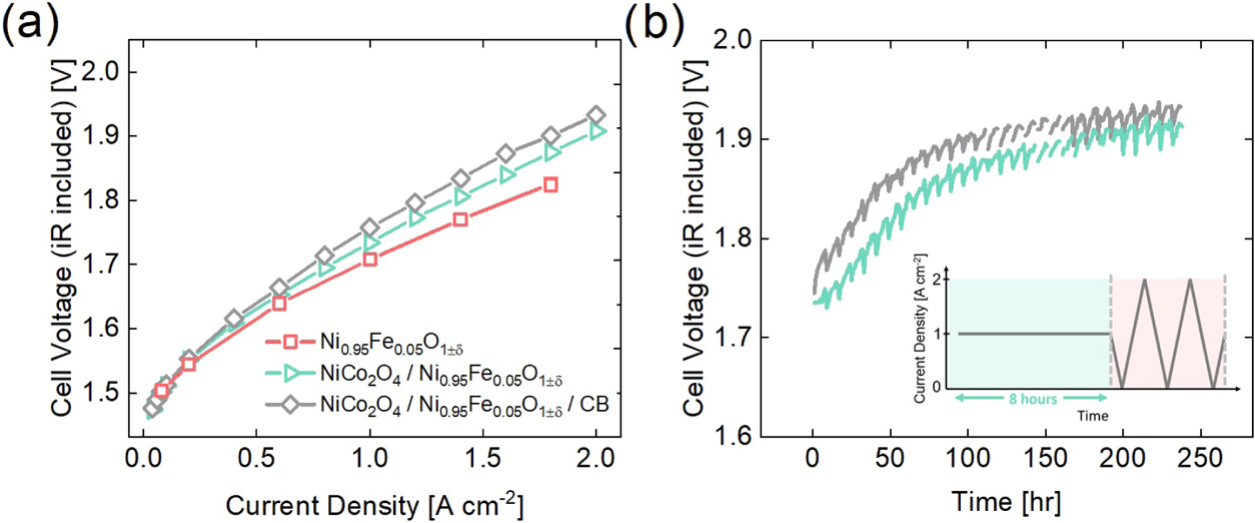
AEMWE measurements of NiFe and composites. (a) Polarization curve. (b) Stability measurement. A constant current density of 1 A cm^–2^ was applied for 8 h, followed by increasing the current density stepwise to 2 A cm^–2^ twice with steps of 0.2 A cm^–2^, holding for 30 s at each step. The inset describes the current density over time, repeated for over 200 h. The cell voltage is plotted every hour. Liquid 1 M KOH electrolyte was circulated through the cell.

The stability of the catalysts NiFe/NiCo and NiFe/NiCo/CB in an AEMWE single cell was assessed using a galvanostatic protocol. The protocol, shown schematically in the inset of Figure 3b, consisted of increasing the current density stepwise to 2 A cm^–2^ twice (0.2 A cm^–2^ steps, holding 30 s at each step) and then holding for 8 h at 1 A cm^–2^. This protocol was repeated for over 200 h and the results are shown in Figure 3b. The oscillations in the data are due to the change in current density every 8 h. The initial cell voltage of NiFe/NiCo with and without carbon is relatively similar. However, within the first few h NiFe/NiCo/CB has a sharp increase in cell voltage. From then on, the change in cell voltage over the stability protocol is roughly mirrored in the two samples. Both samples decrease in performance until around 75 h, with a more pronounced degradation for the NiFe/NiCo/CB MEA. After 75 h, the cell voltage becomes relatively constant around 1.9 V, with slightly superior performance for the NiFe/NiCo MEA. Figures S11a,b, and S12–S14 display the cross-section SEM images and corresponding EDX mapping of the NiFe/NiCo/CB MEA and other samples before and after the AEMWE stability test. EDX mapping of the composite MEAs (Figures S12–S14) confirmed the presence of Ni, Co, and Fe, with nickel being the most abundant metal detected. The aluminum signal is attributed to the SEM stubs. Although all expected metals were detected, the elemental ratios showed slight variation from the initial composition, potentially due to metal leaching and subsequent surface composition changes during electrochemical testing, even within the expected EDX error. There is no significant change in the anode catalyst layer when comparing the structures observed in the SEM images of the catalyst powders (Figures S3–S6) and the MEA before the stability tests (Figure S11) with those of the MEA after the stability tests (Figures S12–S14).

Subsequently, the catalysts’ performance in a fuel cell was also assessed through polarization curves in Figure 4a and the Tafel plot is shown in Figure 4b. NiCo (without carbon) was measured as a comparison to demonstrate the difference between a single ORR catalyst and a composite in AEMFC. NiFe/NiCo and NiFe/NiCo/CB were also measured in an AEMFC single-cell setup. However, when attempting to measure NiFe/NiCo without carbon, the cell resistance was too high, even after several repetitions, and the protocol could not proceed. Figure 4a shows that as the current density increases stepwise, the cell voltage for NiCo decreases sharply and linearly. Around 0.04 A cm^–2^ the measurement was stopped due to a sudden drop in cell voltage. In comparison, NiFe/NiCo/CB is able to achieve higher current densities. The Tafel plot in Figure 4b shows that at lower current densities (<0.02 A cm^–2^) NiCo and NiFe/NiCo/CB have the same Tafel slope, while at higher current densities (>0.02 A cm^–2^) NiFe/NiCo/CB is superior. These results suggest that samples without carbon are unable to reach high current densities due to high Ohmic and mass transport losses.

**Figure 4:**
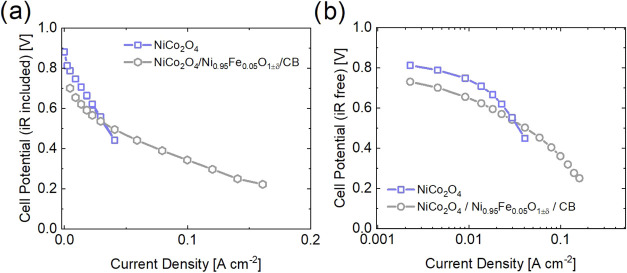
AEMFC (a) polarization curve and (b) Tafel plot of NiCo (without carbon) and NiFe/NiCo/CB. The sample NiFe/NiCo without carbon was unable to be measured due to high resistance.

During AEMFC measurements, a reference electrode was used in order to measure the anode and cathode potential (Figure S18). The cathode potential more clearly shows a significant decrease in the performance of NiCo compared to the other samples. Additionally, NiCo/CB (NiCo with the addition of carbon) and a commercial Pt/CB were also measured as references in Figure S18. NiCo/CB significantly outperforms NiCo and NiFe/NiCo/CB. Therefore, NiCo is the more active ORR catalyst but requires carbon to achieve high current densities. Most likely carbon increases the porosity and gas transport within the catalyst layer. Due to the inability of the samples to achieve high current densities in AEMFC, no stability protocol was performed. Figures S11c,d, and S15–S17 display the cross-section SEM images and corresponding EDX mapping of the NiFe/NiCo/CB MEA and the other samples before and after AEMFC polarization. Similar to the observations after AEMWE operation, there are no significant changes to the anode and cathode catalyst layers visible with SEM after polarization. Similarly, the EDX mapping of the composite MEAs (Figures S15–S17) showed the continued presence of Ni, Co, and Fe, with nickel remaining the most prominent element. Minor deviations in elemental ratios compared to the initial composition may reflect surface changes induced by electrochemical operation, including potential metal leaching.

## Discussion

At the beginning of this study, the aim was to design a high-performing bifunctional catalyst without carbon, due to the occurrence of carbon corrosion at oxidative potentials. The study started with screening a wide range of materials in RDE for their ORR and OER activity, as well as their stability to alternating potentials. The most active samples after stability testing were NiCo and NiFe. Therefore, they were chosen for further investigation as the composite NiFe/NiCo. No sample was found that did not lose significant ORR activity after the alternating potential stability test. Additionally, NiCo was the only sample that had promising ORR activity without carbon in RDE measurements.

The initial performance of NiFe/NiCo without carbon tested as thin, porous catalyst layers in RDE was promising. However, when the catalyst was subsequently tested in a single-cell AEMFC, the performance was severely affected by several issues. NiFe is several orders of magnitude more resistive than NiCo and our results suggest that when NiFe was used without carbon, the catalyst layer was too resistive. However, electrical conductivity is not the sole reason for the catalysts’ poor fuel cell performance. NiCo with metal-like conductivity was unable to reach high current densities, although NiCo/CB was able to. Therefore, carbon is most likely also contributing to enhanced gas permeation in the catalyst layer.^69,70^ Pletcher *et al*.^33^ found that introducing a pore former to NiCo lead to increased ORR performance during alternating testing between ORR and OER due to improved gas transport.

For AEMWE measurements, significant Ohmic and mass transport losses were not encountered because a 1 M KOH liquid electrolyte was used and reactant gas permeability is not required. When carbon was added to the catalyst layers for electrolyzer tests, the performance during stability testing was only slightly impacted. The stability test used here was most likely not long enough to observe the more severe consequences of carbon corrosion.

Carbon plays an important role of providing electrical conductivity pathways throughout the catalyst layer.^50,71,72^ Without a liquid electrolyte, this role becomes even more important. In RDE, thin catalyst layers are utilized and liquid electrolyte is present. Additionally, in RDE the gas permeation properties of the catalyst layer are not probed. Therefore, as shown in this study, the catalyst’s ORR performance in RDE is not always able to predict the catalyst’s behavior in a fuel cell or electrolyzer.

In RDE, the OER activity of all catalysts increased over time, most likely due to the formation of an oxyhydroxide surface layer or similar surface reconstruction. However, during the single-cell electrolyzer stability test, the performance slightly decreased instead. It is most likely that the surface reconstruction had already occurred in the single-cell test during polarization, while in RDE the surface reconstruction was still ongoing during stability testing, possibly due to the alternation between oxidizing and reducing conditions. Indeed, it is also interesting to note that during alternating stability testing, the oxyhydroxide layer continues to grow even though the catalyst is consistently exposed to reducing potentials.

The strategy of combining an ORR catalyst with an OER catalyst in a bifunctional composite electrode is promising but requires further consideration. The NiCo/NiFe catalyst was on par with NiFe for OER activity in RDE and AEMWE. This indicates that the addition of a conductive, but less OER active catalyst does not impede the total OER activity of the composite. More factors need to be considered when discussing the ORR activity of the composite. The initial ORR activity of NiCo/NiFe was less than NiCo in RDE and AEMFC. However, after stability testing in RDE, the composite sample NiCo/NiFe presented higher ORR activity than NiCo. Regardless, both samples, NiCo and NiFe/NiCo, required carbon in the catalyst layer to achieve higher current densities in AEMFC.

For future bifunctional catalyst design, emphasis should be placed on the catalysts’ electrical conductivity and porosity. Although outside of the scope of this paper, alternative methods of catalyst deposition and MEA fabrication can also lead to improved porosity in the catalyst layer.^73–75^ Additionally, further research is needed to design a catalyst that does not lose significant ORR activity when exposed to oxidative potentials or alternating potential cycling. Single-cell testing is essential to understanding how the catalyst will perform in a fuel cell, electrolyzer, or URFC. It should be noted that ideally, the AEMFC and AEMWE single-cell tests would have been performed in a URFC. The next step would be to perform an accelerated stress test in a URFC, alternating between fuel cell and electrolyzer operation.

## Conclusions

This study aimed to understand the key properties and challenges of developing high-performing bifunctional catalysts for ORR and OER. After screening a wide variety of catalysts, NiFe/NiCo was chosen as the most promising composite electrode. Rigorous stability testing in RDE resulted in some degradation of the composite’s ORR activity under URFC conditions, while the OER activity improved. However, the RDE results were not mirrored in AEMFC and AEMWE single-cell MEA testing. Without carbon, the composite experienced fuel cell performance losses due to electrical conductivity and gas permeability issues, properties that were not probed with RDE. In contrast, the composite excelled in AEMWE measurements without carbon. Overall, NiFe/NiCo and composite electrode designs in general are a promising strategy for URFCs, especially for water electrolysis. However, careful consideration is needed for optimizing the composites for fuel cell performance.

## Experimental Method

### Material Synthesis

The material NiCo_2_O_4_ was synthesized by first dissolving 1.40 mmol of cobalt nitrate hexahydrate (98%, Sigma-Aldrich) and 0.70 mmol of nickel nitrate hexahydrate (99.9%, Sigma-Aldrich) in 25 mL of Milli-Q water with 4 mmol of urea. In a Teflon-lined autoclave with a capacity of 50 mL, the mixture was heated at 120 °C for 15 h in an atmosphere muffle furnace. The resulting material was rinsed three times with water, centrifuged, and dried at 80 °C. The spinel was formed by heating the material in an industrial lab oven vertical style with a maximum capacity of 30 L from FORTEX at 450 °C for 4 h. The material Ni_0.95_Fe_0.05_O_1±δ_ was synthesized using a flame-spray method described previously.^47^ The metal nitrate precursors [nickel nitrate hexahydrate (99.9%, Sigma-Aldrich) and iron nitrate nonahydrate (≥98%, Sigma-Aldrich)] were dissolved in acetic acid (≥99.0%, Roth) and Milli-Q water in stoichiometric amounts for a total metal concentration of 0.1 M. In total, it was prepared 1 L of the final solution. Then the solution was pumped through an acetylene flame at a rate of 50 mL min^–1^. The resulting nanoparticles were collected on a filter. Graphitized carbon black (1333-86-4, Sigma-Aldrich) was bought commercially. The specific chemical formulas of materials synthesized were based on the nominal stoichiometry used during synthesis.

### Materials Characterization

The electrical conductivity of the perovskite nanopowders was measured using ex situ 4-wire impedance spectroscopy with an amplitude of 500 mV from a frequency of 1 MHz to 10 mHz. The samples were pressed into a pellet under a pressure of 0.6 MPa for 5 min. Brunauer–Emmett–Teller (BET) analysis of N2 adsorption isotherms (Autosorb-1, Quantachrome Instruments) was performed to find the specific surface area. X-ray diffraction (XRD) was measured on powder samples in Bragg–Brentano mode (Smartlab, Rigaku) with Cu Kα radiation. A Zeiss Supra scanning electron microscope (SEM) was used for imaging the catalysts with a voltage of 5 kV. For the powder catalysts, each sample was dispersed in isopropanol (IPA) using ultrasonic bath sonication (VWR, HF 45 kHz, 50 W, Malaysia), then drop-cast onto an aluminum substrate mounted on SEM stubs. The images were treated using ImageJ. More information can be found in the SI.

### RDE

All RDE measurements were completed in oxygen-saturated 0.1 M KOH (99.99%, Sigma-Aldrich) with a 1600 rpm rotation rate. Cyclic voltammetry was conducted with a scan rate of 5 mV s^–1^. A Hg/HgO reference electrode (0.1 M KOH filled, RE 61AP, ALS) and a gold mesh counter electrode were used. All potentials are Ohmic drop corrected using impedance spectroscopy and are given versus RHE by calibrating the reference against a Pt mesh in hydrogen-saturated electrolyte (0.925 V vs RHE). All currents are normalized by the area of the glassy carbon electrode surface (0.196 cm^2^). The catalyst ink [catalyst (10 mg), Milli-Q water (1.5 mL), isopropanol (1 mL), and Na^+^-exchanged Nafion (20 μL) (5 wt %, Sigma-Aldrich)] was sonicated for 30 min and 15 μL were drop-casted onto the surface of the polished glassy carbon disk. The electrodes were dried in the air, yielding an electrocatalyst loading of 0.30 mg_oxide_ cm^–2^. For the composite NiCo/NiFe, the 10 mg of catalyst consisted of 5 mg of NiCo and 5 mg of NiFe. For the composite with carbon, 2.8 mg of acetylene carbon black was added to the ink.

### AEMFC & AEMWE Single-Cell Testing

The carbon composites had 25 wt % carbon added additionally. The inks were planetary ball milled for 30 min without the ionomer and a further 30 min with the ionomer added. The inks were sprayed onto the membrane using a pulse-swirl-spray (PSS, Nordson Co. Ltd.) technique. Low-pressure hot-pressing (0.2 kN, 80 °C) of the MEA occurred for AEMWE. An ammonium-containing copolymer (QPAF-4) was used for the membrane and ionomer and was synthesized in-house via the procedure outlined previously.^68^ For AEMFC the membrane thickness was 30 μm and for AEMWE it was 50 μm.

For AEMFC, the anode catalyst consisted of Pt/CB (46.9% Pt, TEC10E50E, Tanaka) with an ionomer/carbon ratio of 0.8 and total loading of 0.2 mg_pt_ cm^–2^. The anode gas diffusion layer (GDL) was carbon cloth with microporous layer (MPL) (W1S1010, CeTech). The cathode catalyst loading was 2.0 mg cm^–2^ with an ionomer/catalyst ratio of 0.43 and the cathode GDL was carbon paper with MPL (22BB, Sigracet). The single cell had an area of 4.41 cm^2^, a temperature of 60 °C, and was compressed to 10 kgf cm^–2^. The feeding gases had a flow rate of 100 mL min^–1^ and relative humidity of 100%. The AEMFC setup is explained previously in further detail.^76^ The current density was increased stepwise until a maximum of 1.0 A cm^–2^ and held at each step for 1 min. The cell voltage was monitored as the current density was increased until the limit of approximately 0.2 V was achieved.

For AEMWE, the anode GDL was Ni mesh (1Ni06-020, Bekaert) and the cathode gas diffusion electrode (GDE) was Pt/CB sprayed onto Teflon-treated carbon fiber paper (TGP-H-120, Toray). The anode had a catalyst loading of 2.0 mg cm^–2^ and an ionomer/catalyst ratio of 0.15 for initial NiFe testing and for all other measurements a ratio of 0.43 was used to mirror that of AEMFC. The cathode catalyst loading was 1 mg_pt_ cm^–2^ and the ionomer/carbon ratio was 0.6. The single cell had an area of 1 cm^2^, a temperature of 80 °C, and was compressed to 0.75 MPa. Liquid 1 M KOH electrolyte at 80 °C was recirculated with a flow rate of 10 mL min^–1^ after flowing through a 0.6 mm Teflon mesh filter. The catalyst was activated by increasing the current density twice to 1 A cm^–2^ with steps of 0.2 A cm^–2^, holding for 30 s at each step, then twice to 2 A cm^–2^. Then, a constant current density of 1 A cm^–2^ was applied for 8 h followed by increasing the current density stepwise to 2 A cm^–2^ twice. This procedure was repeated for over 200 h. Detailed experimental information on the single-cell tests is found in the Supporting Information.

## Supplementary Material



## Notes

Detailed experimental procedures for membrane and binder preparation, catalyst layer fabrication, AEMFC and AEMWE testing, and SEM/EDX and XPS analysis; RDE screening data; XRD patterns and XPS spectra of catalysts; cross-section SEM images with EDX mapping; polarization curves for AEMFC and AEMWE (PDF)
