# Supplementary material for: Composite Bifunctional Electrocatalyst for the Oxygen Reduction and Evolution Reactions
Source: ACS Mater Au. 2025 Jul 3;5(5):798–808. doi: 10.1021/acsmaterialsau.5c00034 (PMC12426780; doi:10.1021/acsmaterialsau.5c00034)
Supplement: Supplementary file 1 [file mg5c00034_si_001.pdf]

Supplementary Information

Composite bifunctional electrocatalyst for the oxygen reduction and evolution reactions

Casey E. Beall[1], Emiliana Fabbri[1,*], Juliana Bruneli Falqueto[1,*], Sebastian Siegrist[1,2], Jinzhen Huang[1], Natasha Hales[1], Dominika Baster[1], Mario El Kazzi[1], Sayaka Takahashi[3], Yuto Shirase[3], Makoto Uchida[3], Thomas J. Schmidt[1,2]

Corresponding Author

* Dr. Emiliana Fabbri, emiliana.fabbri@psi.ch

* Dr. Juliana Bruneli Falqueto, juliana.bruneli-falqueto@psi.ch

Author Affiliations

[1]PSI Center for Energy and Environmental Science, 5232 Villigen PSI, Switzerland

[2]Institute for Molecular Physical Science, ETH Zürich, 8093 Zürich, Switzerland

[3]Hydrogen and Fuel Cell Nanomaterials Center, University of Yamanashi, 400-0021, Kofu, Japan

Experimental Method

Membrane and Binder

An ammonium containing copolymer (QPAF-4) was used for the membrane and binder due to QPAF-4's high hydroxide ion conductivity and excellent mechanical properties and chemical stability.[1] QPAF-4 was synthesized in-house via the procedure outlined by Ono et al.[1]

An ion exchange capacity (IEC) of 1.5 was used for all measurements except for preliminary $NiCo_2O_4$ (NiCo) AEMFC measurements without any conductive additives, where a ratio of 2.0 was used. For AEMFC the membrane thickness was 30 µm and for AEMWE it was 50 µm. Ion exchange of the membrane and binder were also completed on all measurements except for preliminary $Ni_{0.95}Fe_{0.05}O_{1\pm\delta}$ (NiFe) AEMWE measurements without any conductive additives. The membrane and the binder were ion exchanged over a period of a few days. First, they were immersed in 1.0 M KOH at 80 °C for 2 days. Then they were rinsed with water and soaked in 0.5 M KCO_3 for one day. Lastly, the solution was exchanged with Milli-Q water for one more day. Then, QPAF-4 was dried by pressing between two cloths. The same binder was used for the AEMFC anode and cathode and AEMWE anode.

Catalyst Layer Preparation

For AEMFC, both anode and cathode inks were made with the following ratio of materials: 0.3 g catalyst, 3.75 g of Milli-Q water, 5.1 g of methanol, and 2.6 g binder solution (5 wt% QPAF-4 in methanol). For AEMWE, the anode ink consisted of the following ratio of materials: 1 g catalyst, 12.6 g of Milli-Q water, 17 g of methanol, and 3 g binder solution (5 wt% QPAF-4 in methanol). The composites NiCo/CB consisted of 25 wt% conductive additive. The composite NiCo/NiFe consisted of 50 wt% of both catalysts. The composite NiCo/NiFe/CB (CB being carbon black) consisted of 25 wt%. The inks were planetary ball milled with 20 zirconia beads (5 mm diameter) for 30 min without the binder solution and a further 30 min with the addition of the filtered QPAF-4-MeOH binder solution. Then, the inks were mixed for at least 12 hours before spraying. The inks were sprayed onto the membrane using a pulse-swirl-spray (PSS, Nordson Co. Ltd.) technique. No pressing or hot-pressing

of the membrane electrode assembly (MEA) was conducted for AEMFC. Low-pressure hot-pressing (0.2 kN, 80 °C) of the MEA occurred for AEMWE.

AEMFC

The samples were tested in an AEMFC set up with an area of 4.41 cm^2 (2.1 cm x 2.1 cm). The cell temperature was 60 °C and the feeding gases were controlled by a mass flow rate controller (100 mL min^{-1}) and humidified to a relative humidity of 100% by bubbling through a hot water reservoir. Silicone/poly(ethyl benzene-1, 4-dicarboxylat)/silicone gaskets (SB50A1P, Maxell Kureha Co., Ltd.) with a thickness of 200 µm and serpentine flow graphite plates were used on both the anode and cathode side and the cell was compressed to a pressure 10 kgf cm^{-2} with four springs. No back pressure was applied to the anode and cathode sides.

The anode catalyst consisted of Pt/CB (46.9% Pt, TEC10E50E, Tanaka) with a QPAF-4 binder/carbon ratio of 0.8 and total loading of 0.2 mg_{pt} cm^{-2}. The anode gas diffusion layer (GDL) was carbon cloth with MPL (W1S1010, CeTech). The cathode catalyst loading was 2.0 mg cm^{-2} with a QPAF-4 binder/catalyst ratio of 0.43 and the cathode GDL was carbon paper with MPL (22BB, Sigracet). The anode and cathode potential vs. the reversible hydrogen electrode (RHE) were measured by a reference electrode on the cathode side. Hydrogen was supplied to the reference electrode from the anode outlet and heated to 90 °C. A 5 cm diameter disk of GDE (GDL from cathode, sprayed with Pt/CB ink) was applied to the reference electrode, where the CL was in contact with the membrane and the GDL contacted a gold wire, which was connected to the anode and cathode. The electrodes were connected to a multi-input data logger (NR-500, KEYENCE Corp.) and a high voltage measurement unit (NR-HV04, KEYENCE Corp.). The AEMFC set-up is explained previously in further detail.[2]

The current density was increased stepwise until a maximum of 1.0 A cm^{-2} and held at each step for one minute. The cell voltage was monitored as the current density was increased until the limit of approximately 0.2 V was achieved. Then, the current density was subsequently decreased in the same manner. This was repeated until the cell performance no longer increased. The measurement was monitored using an electronic load (PLZ664WA and KFM2150, Kikusui Electronics Corp.) and

controlled by a measurement system (fuel cell characteristic evaluation device, Netsuden Kogyo Corp.) The resistance for iR correction was determined from alternating current (AC) impedance (KFM2150, Kikusui Electronics Corp.) for current densities above 0.1 A cm^{-2}. Below this value, resistances are difficult to measure; therefore, measured with a 1 kHz external resistance meter (MODEL 3566, Tsuruga Electric Corp.) was used instead.

AEMWE

A cell with an area of 1 cm^2 (1 cm x 1 cm) was used for AEMWE measurements. The cell was operated at a temperature of 80 °C and compressed to a pressure of 0.75 MPa. The 1 M KOH electrolyte was preheated to 80 °C and was recirculated with a flow rate of 10 mL min^{-1} after flowing through a 0.6 mm Teflon mesh filter with 5 cm diameter. Approximately 7 L of electrolyte were recirculated and controlled with a mass flow controller (8500MM-0-2-1, Kofloc).

EPDM gaskets (Chemix) with 200 µm thickness (anode) and 300 µm thickness (cathode) were employed. The anode GDL was Ni mesh (1Ni06-020, Bekaert) and the cathode gas diffusion electrode (GDE) was Pt/CB sprayed onto Teflon treated carbon fiber paper (TGP-H-120, Toray). The anode had a catalyst loading of 2.0 mg cm^{-2}. The anode catalyst layer had a QPAF-4 binder/catalyst ratio of 0.15 for initial NiFe testing and for all other measurements a ratio of 0.43 was used to mirror that of AEMFC. The cathode catalyst loading was 1 mg_{pt} cm^{-2} and the QPAF-4 binder/carbon ratio was 0.6. Ni separator plates and a gold plated Cu feeding plate were used. AEMWE cell from Chemix by Yokohama National University.

The measurement was controlled using a DC power supply (PWR401L, Kikusui) and by a measurement system (electrolyzer characteristic evaluation device, Netsuden Kogyo Corp.). First, the catalyst was activated by increasing the current density twice to 1 A cm^{-2} with steps of 0.2 A cm^{-2}, holding for 30 s at each step, then twice to 2 A cm^{-2}. After activation, a constant current density of 1 A cm^{-2} was applied for 8 hours followed by increasing the current density stepwise to 2 A cm^{-2} twice. This procedure of holding at 1 A cm^{-2} and increasing twice to 2 A cm^{-2} was repeated for over 200 hours.

SEM/EDX and XPS

A Zeiss Supra scanning electron microscope (SEM) was used for imaging the catalysts with a voltage of 5 kV. For the powder catalysts, each sample was dispersed in isopropanol (IPA) using ultrasonic bath sonication (VWR, HF 45 kHz, 50W, Malaysia), then drop-cast onto an aluminum substrate mounted on SEM stubs. The images were treated using ImageJ. The elemental surface composition was analyzed using energy-dispersive X-ray (EDX) measurements performed with a windowless silicon drift detector (Oxford Ultim Extreme). The error range for atomic percentage measurements is ±2–5% for O and C elements and ±1–2% for the transition metals Co, Fe, Ni, and Al. The aluminum signal originates from the substrate.

The X-ray photoelectron spectroscopy (XPS) spectra were acquired with a VG ESCALAB 220iXL spectrometer (Thermo Fisher Scientific) calibrated on a clean silver surface by measuring the Ag $3d_{5/2}$ peak at a binding energy (BE) of 368.25 eV with a full width at half-maximum (FWHM) of 0.78 eV at a pass energy of 30 eV. Focused monochromatized Al Kα radiation (1486.6 eV). The beam size was ~500 μm^2. The pressure in the analysis chamber was approximately $2 \cdot 10^{-9}$ mbar. All survey spectra were recorded with a dwell time of 10 ms, using the pass energy of 70 eV in steps of 0.5 eV. The spectra acquired in a narrow energy scan were recorded using a pass energy of 20 eV in steps of 50 meV and a dwell time of 50 ms. All spectra were calibrated relative to the carbon C 1s peak at 284.8 eV to correct for charging effects. XPS measurements were performed directly on powder samples ground with 10 wt% carbon black in an agate mortar to ensure sufficient conductivity. As a result, only the carbon-containing samples (NiFe, NiCo, and NiFe/NiCo/CB) were analyzed, while the NiFe/NiCo sample without carbon could not be measured.

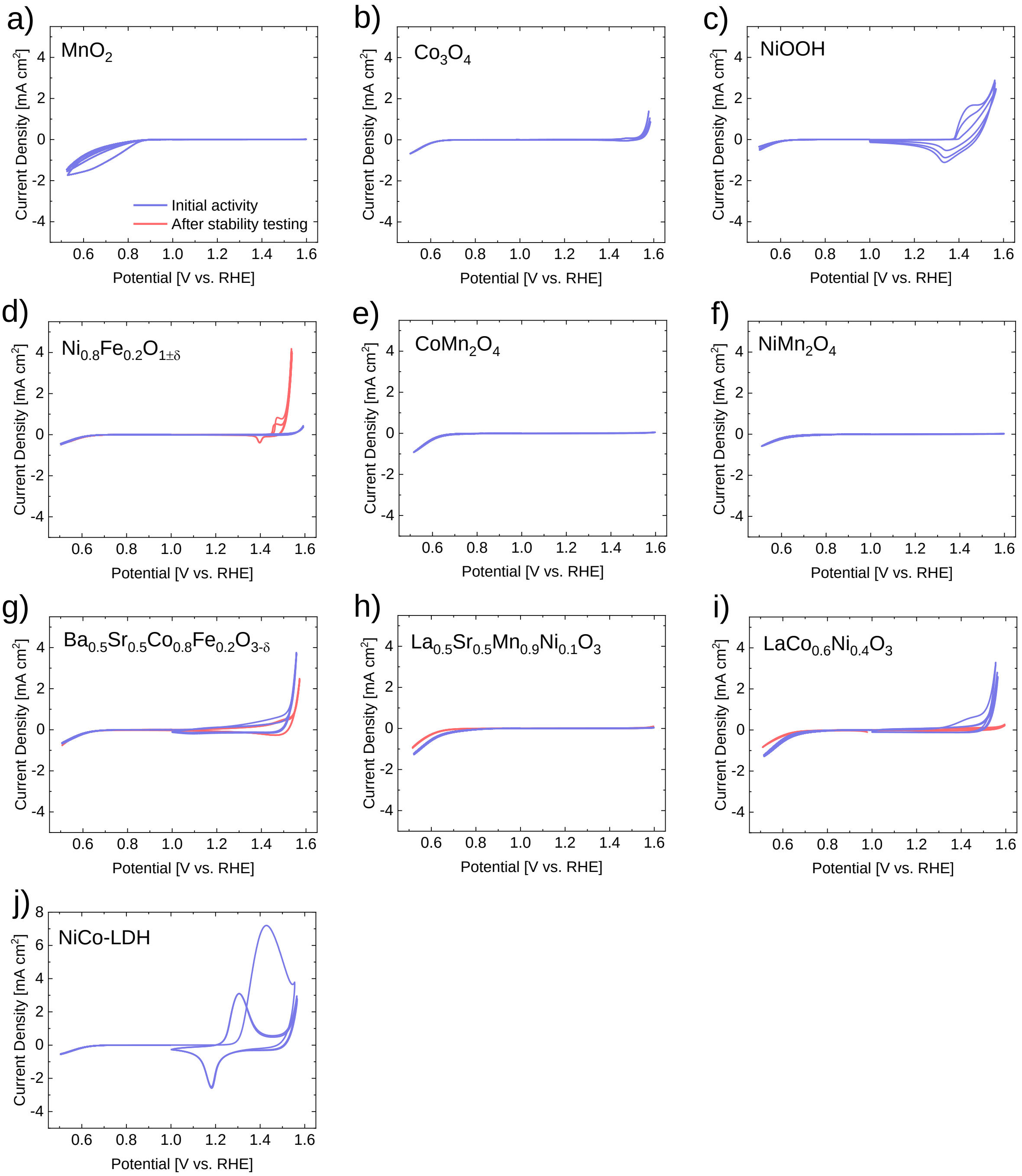

Figure S1. RDE measurements of the ORR and OER activities of Co, Mn, Ni, and Fe containing oxides. Prior to choosing samples for this study, a wide range of non-noble metal oxides were tested for bifunctional activity. Three CVs were performed from 1.0 – 0.5 V (ORR), followed by three CVs from 1.0 – 1.6 V (OER). For all samples the initial activities are given. For select samples the activity is given after stability testing. The stability testing is the same alternating chronoamperometry protocol as in Figure 2a.

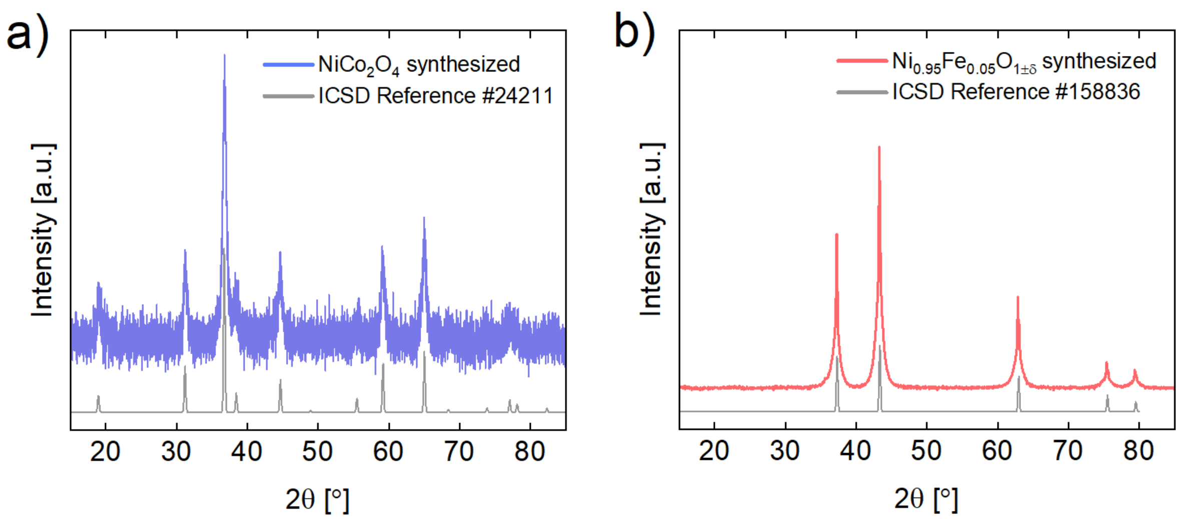

Figure S2. X-ray diffraction (XRD) patterns of **a**) NiCo synthesized by hydrothermal method and calcination and **b**) NiFe synthesized by flame-spray synthesis. The patterns are compared to ICSD database (FIZ Karlsruhe) references.

Table S1. Results from fitting the low frequency range of ex situ 4 wire impedance spectroscopy measurements. Nanopowder samples were measured without carbon. It should be noted that the absolute conductivity values of these measurements should be taken with caution. They are merely meant as a comparison between samples using the same experimental conditions.

	Conductivity [S cm^{-1}]
NiFe	2.8×10^{-7}
NiCo	0.46

SEM and EDX of the catalyst powders and composites. It is important to note that the presence of aluminum (Al) originates from the SEM sample stub and the error percentage for the EDX is ± 2% atomic percentage for Ni, Co, Al, and Fe and ± 5% for C, and O elements.

- NiCo

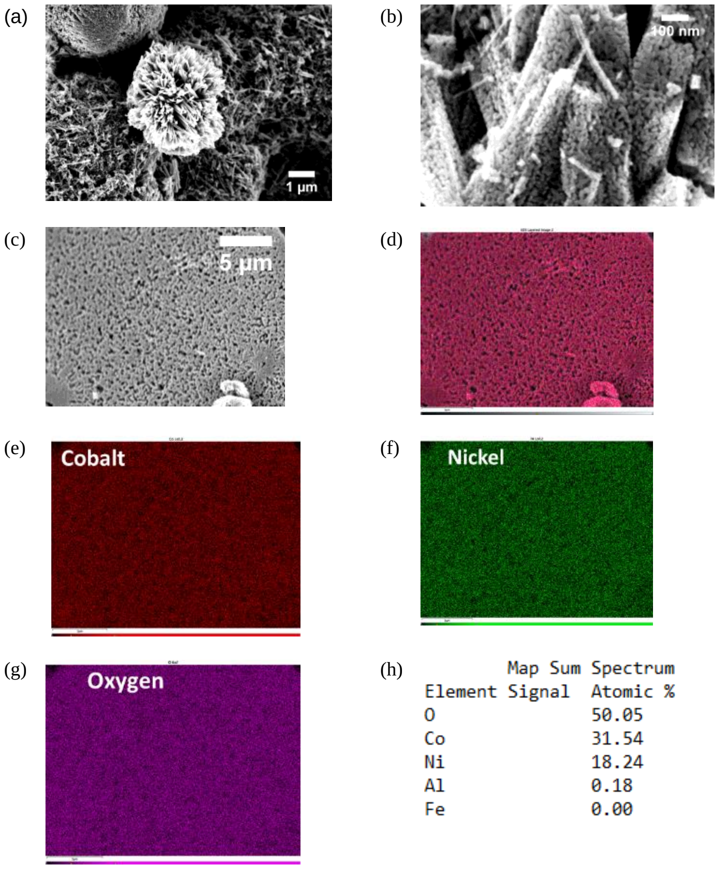

Figure S3. NiCo catalyst powder (**a**) and (**b**) SEM images in high magnification. (**c**) SEM image with lower magnification and the (**d**) corresponding EDX resulting mapping. Mapping elements: (**e**) cobalt, (**f**) nickel, (**g**) oxygen, and (**h**) atomic composition percentage.

- NiFe

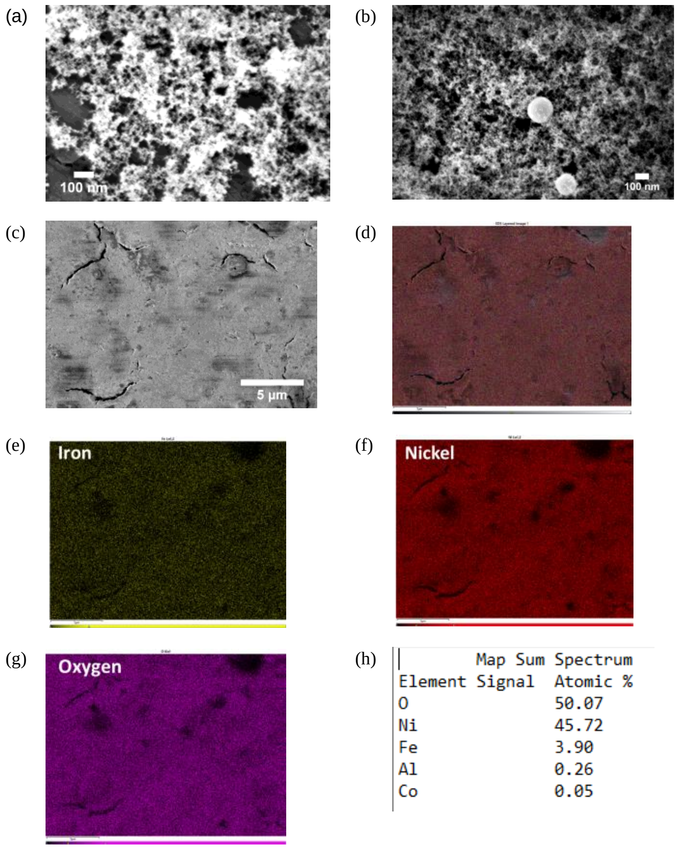

Figure S4. NiFe catalyst powder (**a**) and (**b**) SEM images in high magnification. (**c**) SEM image with lower magnification and the (**d**) corresponding EDX resulting mapping. Mapping elements: (**e**) iron, (**f**) nickel, (**g**) oxygen, and (**h**) atomic composition percentage.

- NiCo/NiFe

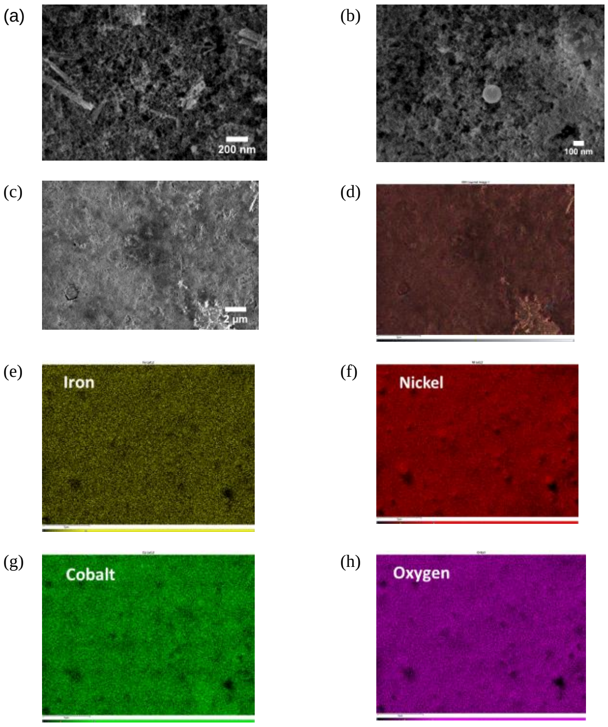

(i)

Element	Map Sum Spectrum Signal	Atomic %
O		50.05
Ni		38.11
Co		9.30
Fe		2.35
Al		0.20

Figure S5. NiCo/NiFe catalyst composite (**a**) and (**b**) SEM images in high magnification. (**c**) SEM image with lower magnification and the (**d**) corresponding EDX resulting mapping. Mapping elements: (**e**) iron, (**f**) nickel, (**g**) cobalt, (**h**) oxygen, and (**i**) atomic composition percentage.

- NiCo/NiFe/CB

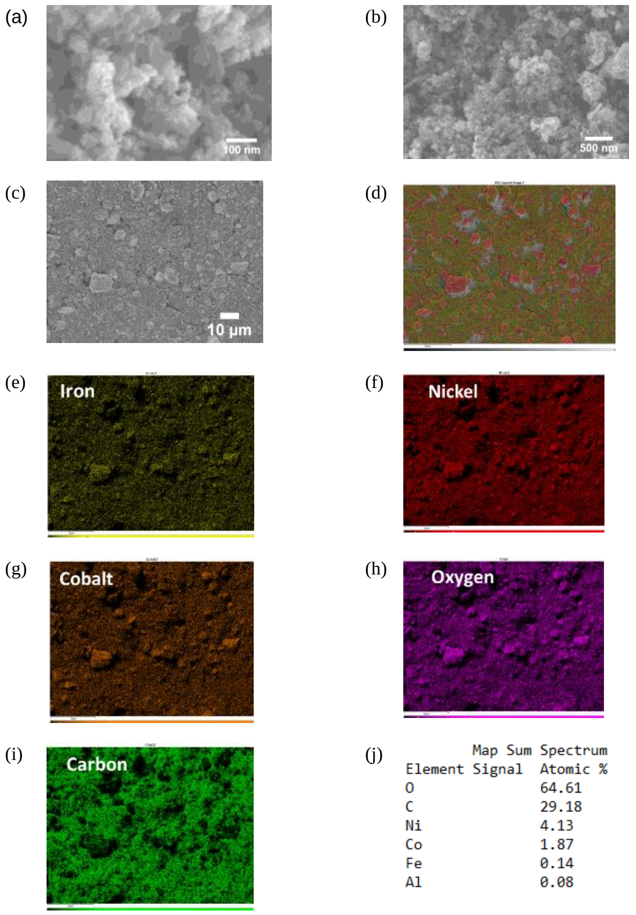

Figure S6. NiCo/NiFe/CB catalyst composite (**a**) and (**b**) SEM images in high magnification. (**c**) SEM image with lower magnification and the (**d**) corresponding EDX resulting mapping. Mapping elements: (**e**) iron, (**f**) nickel, (**g**) cobalt, (**h**) oxygen, (**i**) carbon and (**j**) atomic composition percentage.

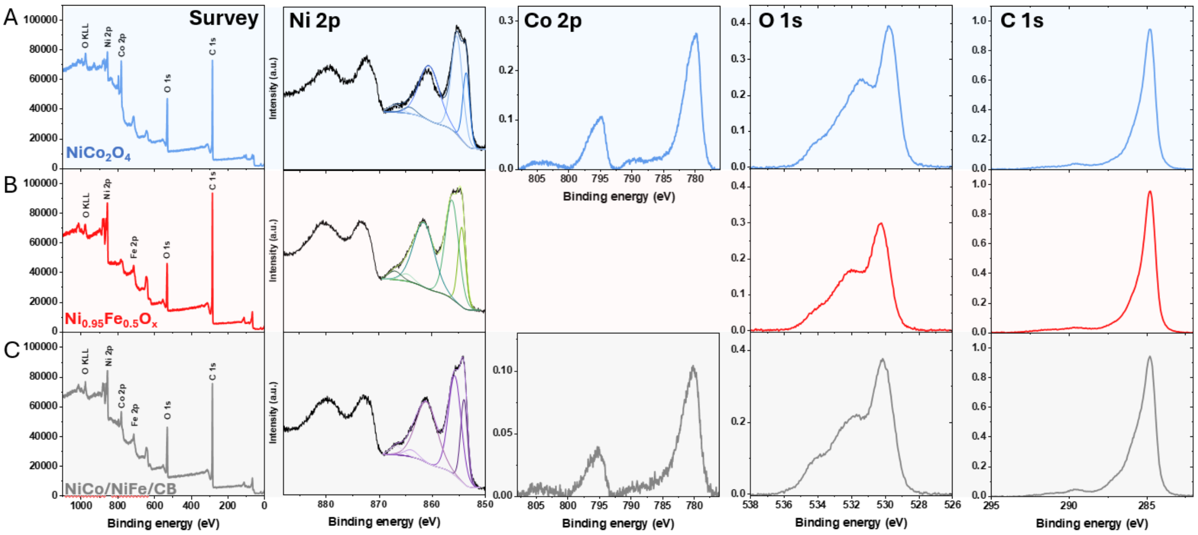

Figure S7. Survey, Ni 2p, Co 2p, O 1s and C 1s XPS spectra of **a**) NiCo, **b**) NiFe and **c**) NiCo/NiFe/CB samples.

Table S2. Spectral fitting parameters for Ni 2p3/2: binding energy (eV), percentage of total area, FWHM value (eV) for pass energy of 20 eV.

Compound	Peak 1 (eV)	%	Peak 1, FWHM, 20 eV Pass	Peak 2 (eV)	%	Peak 2, FWHM, 20 eV Pass	Peak 3 (eV)	%	Peak 3, FWHM, 20 eV Pass
**$NiFe_2O_4$ [Ref (3)]**	854.50	17.30	1.36	856.00	38.20	2.98	861.40	38.50	4.50
**NiFe [This work]**	854.38	17.34	1.67	856.23	38.25	2.97	861.40	38.43	4.59
**NiCo [This work]**	853.67	17.34	1.54	855.44	38.25	2.47	860.60	38.43	4.50
**NiCo/NiFe/ CB [This work]**	854.09	17.34	1.52	855.81	38.25	2.84	861.13	38.43	4.73

Table S3. [Continuation of Table S2] Spectral fitting parameters for Ni 2p3/2: binding energy (eV), percentage of total area, FWHM value (eV) for pass energy of 20 eV.

**Compound**	**Peak 4 (eV)**	**%**	**Peak 4, FWHM, 20 eV Pass Energy**	**Peak 5 (eV)**	**%**	**Peak 5, FWHM, 20 eV Pass Energy**
**$NiFe_2O_4$ [Ref (3)]**	864.70	2.80	3.01	867.0	3.20	2.66
**NiFe [This work]**	864.76	2.79	2.85	866.99	3.18	2.85
**NiCo [This work]**	864.29	2.79	2.80	866.00	3.18	2.80
**NiCo/NiFe/CB [This work]**	864.00	2.79	2.95	866.77	3.18	2.65

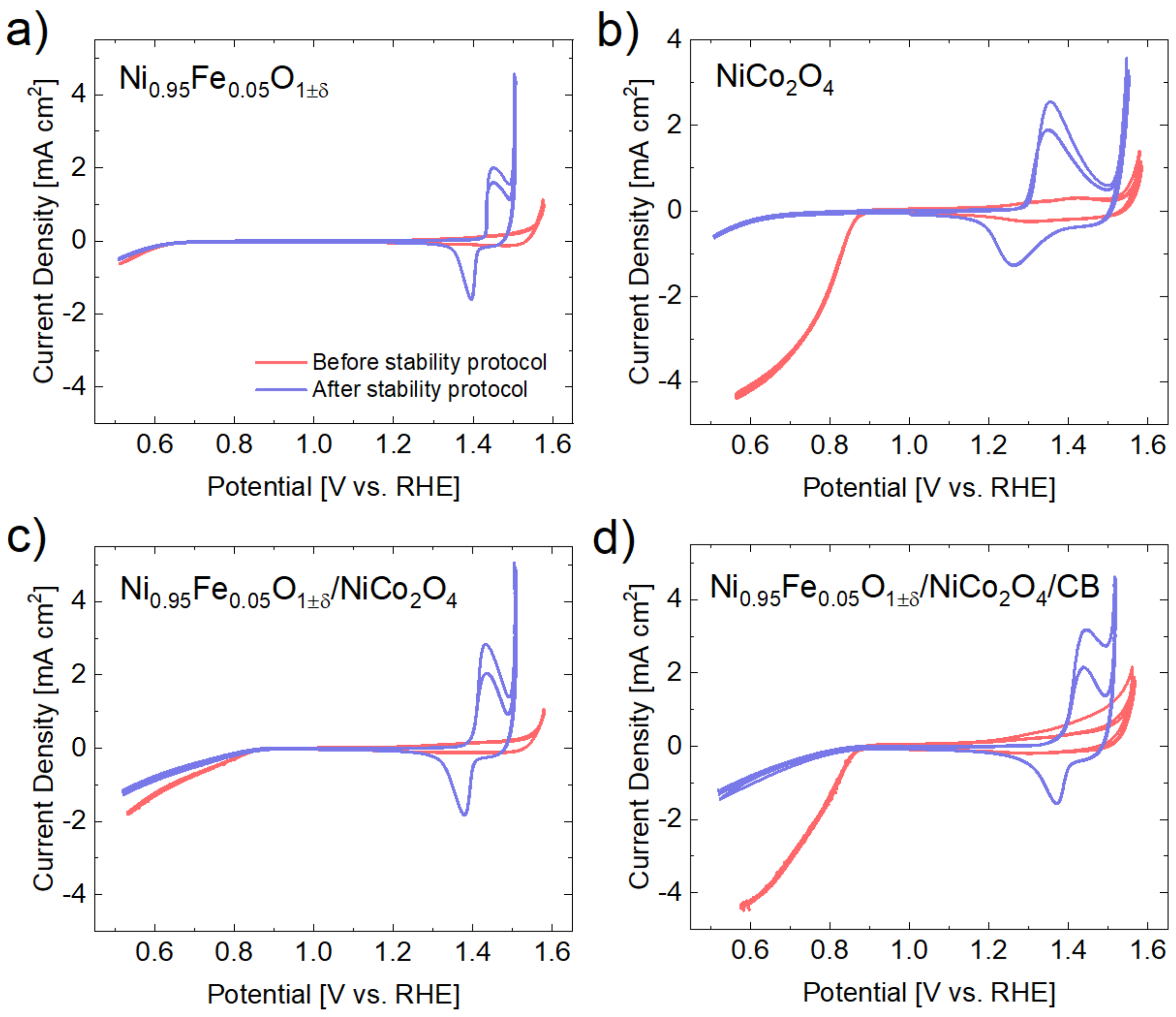

Figure S8. Cyclic voltammetry from RDE measurements before and after stability protocol. **a**) NiFe **b**) NiCo **c**) NiFe/NiCo **d**) NiFe/NiCo/CB

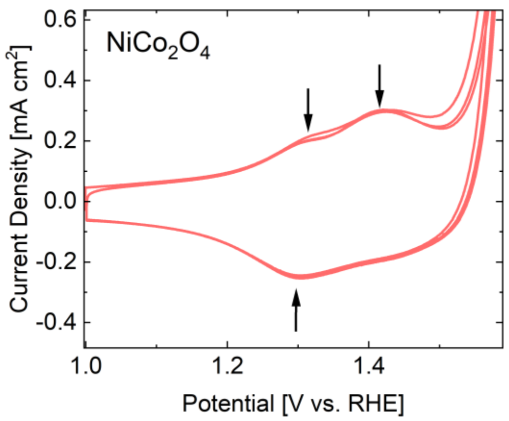

Figure S9. Redox activity for the sample NiCo measured with RDE before stability protocol. Three CVs were performed between 1 - 1.6 V in 0.1 M KOH and 1600 rpm. The same data is shown in Figure 1c and Figure S3b; however, a higher zoom is used here to more clearly see the redox peaks.

Although IrO_2 is typically used as a benchmark in acidic electrolytes, it is also employed in alkaline OER studies due to its well-known catalytic properties.[4] It was therefore selected as a reliable reference for comparison with the synthesized catalysts under alkaline conditions.

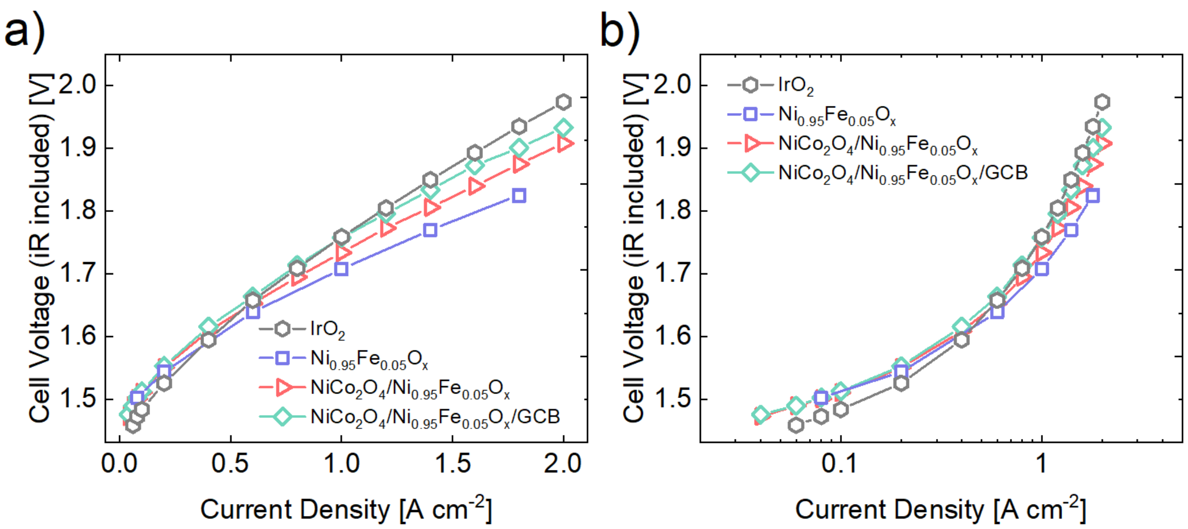

Figure S10. **a**) Polarization curve and **b**) Tafel plot of AEMWE measurements compared to a IrO_2 reference.

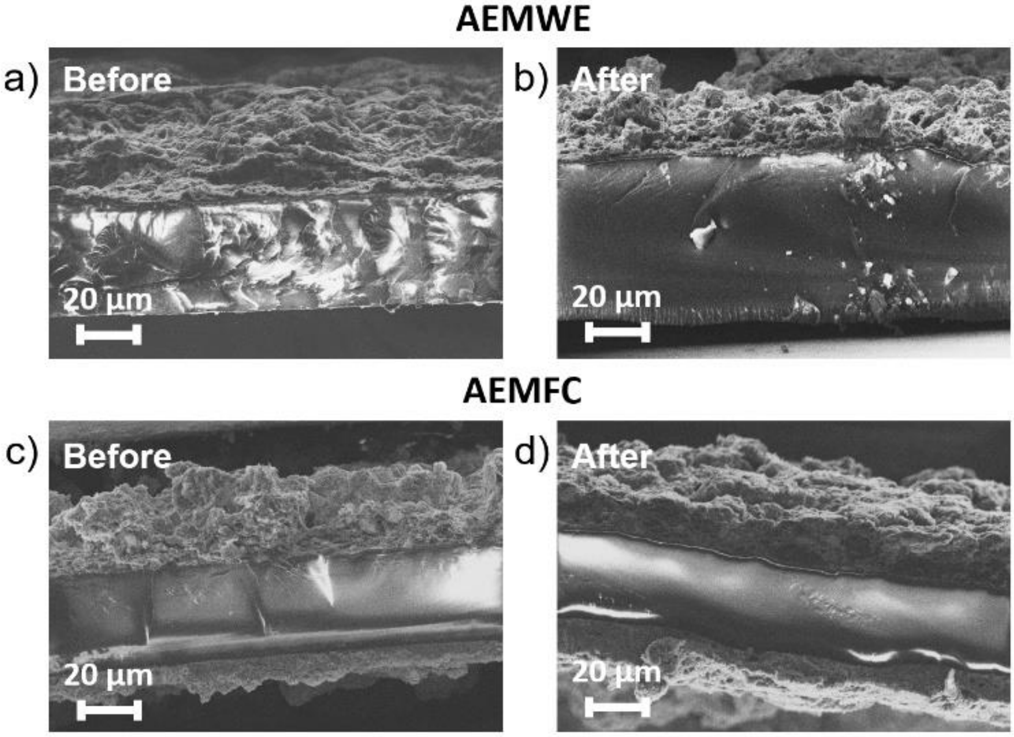

Figure S11. Cross-section SEM images (5 kV) of NiFe/NiCo/CB MEA before and after AEMWE and AEMFC measurements. The cross-sectional cut was made by liquid nitrogen cryogenic fracture. The AEMWE images were taken **a**) before and **b**) after stability measurement. The anode catalyst layer is on top. The AEMFC images were taken **c**) before and **d**) after polarization. The cathode catalyst layer is on top. The thickness of the anion exchange membrane seems to increase after AEMWE measurements. However, understanding the mechanism behind membrane degradation during the OER AEMWE stability test is out of the scope of the present study.

SEM and EDX cross-section analysis after AEMFC and AEMWE stability measurements. The cross-sectional cut was made by liquid nitrogen cryogenic fracture. The atomic percentages obtained from the EDX mapping indicate that the ratios of transition metals Co/Ni and Fe/Ni are consistent with the stoichiometric values from the synthesis of the catalyst powders $NiCo_2O_4$ and $Ni_{0.95}Fe_{0.05}O_1$±δ, as well as with the expected values for the mixed catalyst composites.

It is important to note that the presence of aluminum (Al) originates from the SEM sample stub.

1. AEMWE NiCo/NiFe/CB after

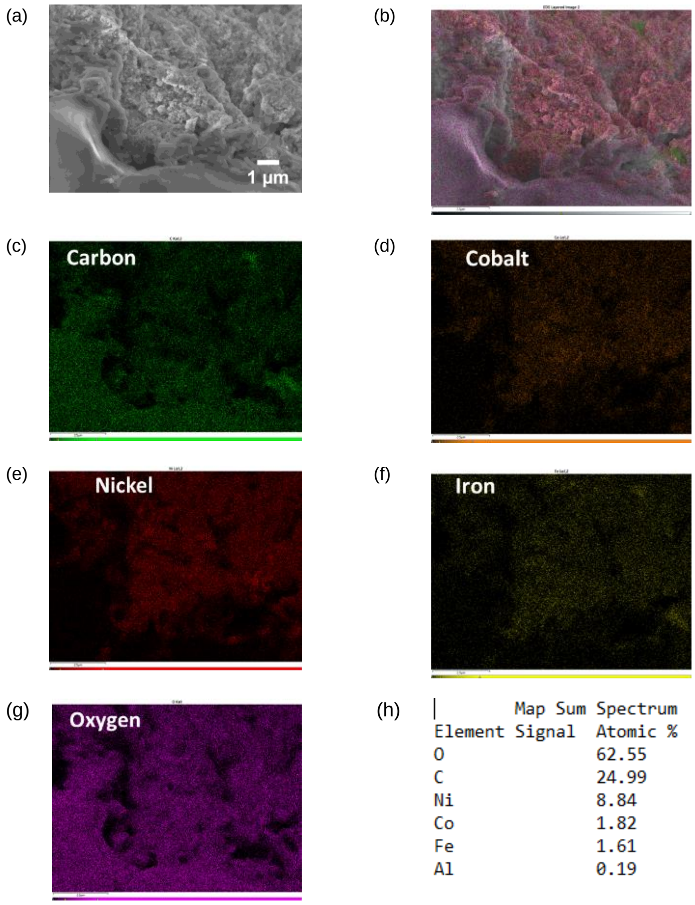

Element	Map Sum Spectrum Signal	Atomic %
O		62.55
C		24.99
Ni		8.84
Co		1.82
Fe		1.61
Al		0.19

Figure S12. MEA of NiCo/NiFe/CB after stability test cross-section (**a**) SEM and (**b**) corresponding EDX resulting mapping. Mapping elements: (**c**) carbon, (**d**) cobalt, (**e**) nickel, (**f**) iron, (**g**) oxygen, and (**h**) atomic composition percentage.

2. AEMWE NiFe after

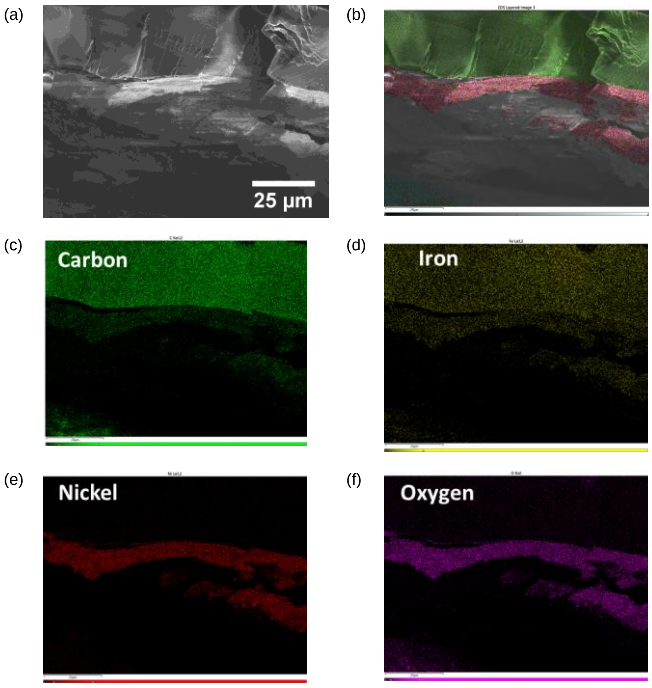

(g)

Element	Map Sum Spectrum Signal Atomic %
O	64.42
C	28.23
Fe	3.94
Ni	2.20
Al	1.21
Co	0.00

Figure S13. MEA of NiFe after stability test cross-section (**a**) SEM and (**b**) corresponding EDX resulting mapping. Mapping elements: (**c**) carbon, (**d**) iron, (**e**) nickel, (**f**) oxygen, and (**g**) atomic composition percentage.

3. AEMWE NiCo/NiFe after

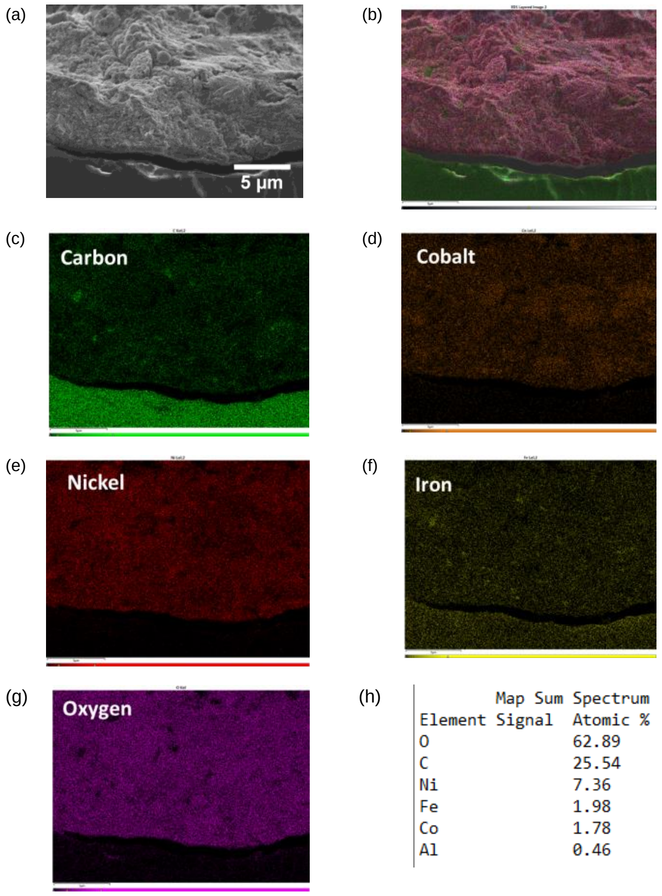

Figure S14. MEA of NiCo/NiFe after stability test cross-section (**a**) SEM and (**b**) corresponding EDX resulting mapping. Mapping elements: (**c**) carbon, (**d**) cobalt, (**e**) nickel, (**f**) iron, (**g**) oxygen, and (**h**) atomic composition percentage.

4. AEMFC NiCo/NiFe/CB after

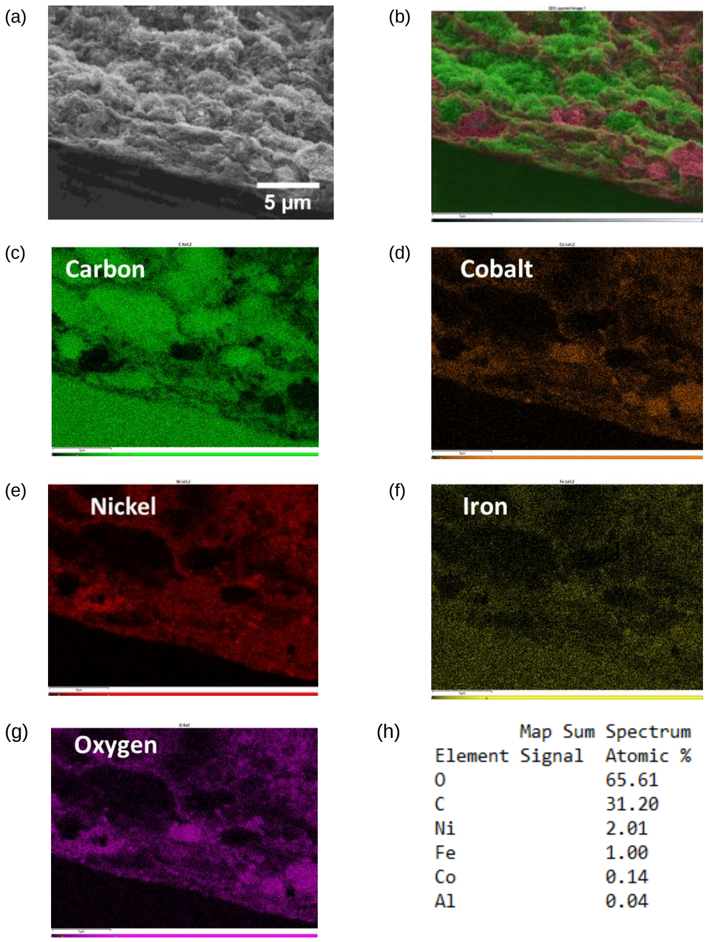

Figure S15. MEA of NiCo/NiFe/CB after stability test cross-section (**a**) SEM and (**b**) corresponding EDX resulting mapping. Mapping elements: (**c**) carbon, (**d**) cobalt, (**e**) nickel, (**f**) iron, (**g**) oxygen, and (**h**) atomic composition percentage.

5. AEMFC NiCo after

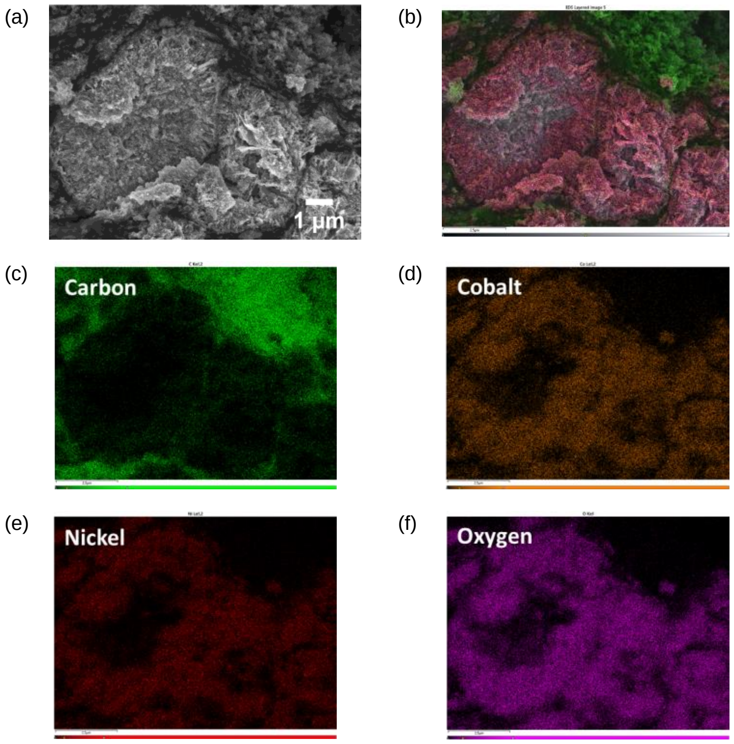

(g)

	Map Sum	Spectrum
Element	Signal	Atomic %
O		62.87
C		25.73
Co		7.17
Ni		3.24
Fe		0.94
Al		0.04

Figure S16. MEA of NiCo after stability test cross-section (**a**) SEM and (**b**) corresponding EDX resulting mapping. Mapping elements: (**c**) carbon, (**d**) cobalt, (**e**) nickel, (**f**) oxygen, and (**g**) atomic composition percentage.

6. AEMFC NiCo/CB after

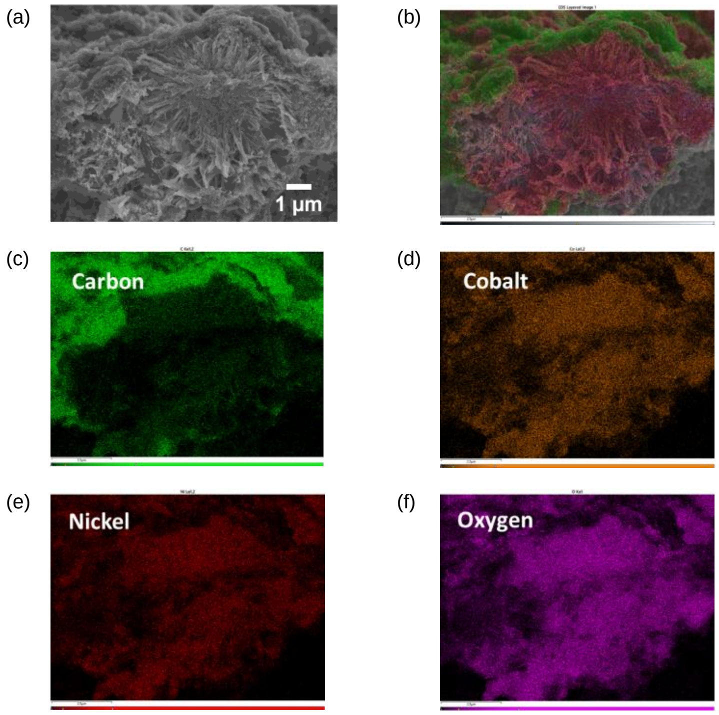

(g)

Element	Map Sum Spectrum Signal Atomic %
O	63.18
C	26.28
Co	6.78
Ni	3.25
Fe	0.34
Al	0.16

Figure S17. MEA of NiCo/CB after stability test cross-section (**a**) SEM and (**b**) corresponding EDX resulting mapping. Mapping elements: (**c**) carbon, (**d**) cobalt, (**e**) nickel, (**f**) oxygen, and (**g**) atomic composition percentage.

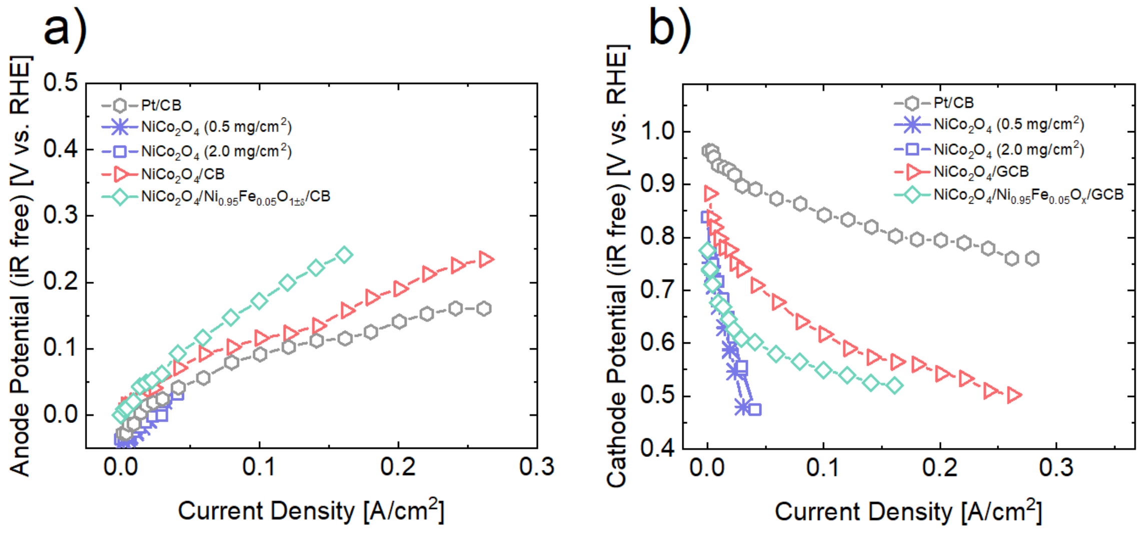

Figure S18. Polarization curves of AEMFC measurements compared to a Pt/CB reference. **a**) Anode potential. **b**) Cathode potential.

References

(1) Ono, H.; Kimura, T.; Takano, A.; Asazawa, K.; Miyake, J.; Inukai, J.; Miyatake, K. Robust Anion Conductive Polymers Containing Perfluoroalkylene and Pendant Ammonium Groups for High Performance Fuel Cells. *J. Mater. Chem. A* **2017**, *5* (47), 24804–24812.

(2) Otsuji, K.; Shirase, Y.; Asakawa, T.; Yokota, N.; Nagase, K.; Xu, W.; Song, P.; Wang, S.; Tryk, D. A.; Kakinuma, K.; Inukai, J.; Miyatake, K.; Uchida, M. Effect of Water Management in Membrane and Cathode Catalyst Layers on Suppressing the Performance Hysteresis Phenomenon in Anion-Exchange Membrane Fuel Cells. *J. Power Sources* **2022**, *522*, 230997.

(3) Biesinger, M. C.; Payne, B. P.; Grosvenor, A. P.; Lau, L. W. M.; Gerson, A. R.; Smart, R. St. C. Resolving Surface Chemical States in XPS Analysis of First Row Transition Metals, Oxides and Hydroxides: Cr, Mn, Fe, Co and Ni. *Appl. Surf. Sci.* **2011**, *257*, 2717.

(4) McCrory, C. C. L.; Jung, S.; Ferrer, I. M.; Chatman, S. M.; Peters, J. C.; Jaramillo, T. F. Benchmarking Hydrogen Evolving Reaction and Oxygen Evolving Reaction Electrocatalysts for Solar Water Splitting Devices. *J. Am. Chem. Soc.* **2015**, *137* (13), 4347–4357.
